# Interference effects in BSM processes with a generalised narrow-width approximation

**DOI:** 10.1140/epjc/s10052-015-3472-z

**Published:** 2015-06-09

**Authors:** Elina Fuchs, Silja Thewes, Georg Weiglein

**Affiliations:** DESY, Deutsches Elektronen-Synchrotron, Notkestr. 85, 22607 Hamburg, Germany

## Abstract

A generalisation of the narrow-width approximation (NWA) is formulated which allows for a consistent treatment of interference effects between nearly mass-degenerate particles in the factorisation of a more complicated process into production and decay parts. It is demonstrated that interference effects of this kind arising in BSM models can be very large, leading to drastic modifications of predictions based on the standard NWA. The application of the generalised NWA is demonstrated both at tree level and at one-loop order for an example process where the neutral Higgs bosons *h* and *H* of the MSSM are produced in the decay of a heavy neutralino and subsequently decay into a fermion pair. The generalised NWA, based on on-shell matrix elements or their approximations leading to simple weight factors, is shown to produce UV- and IR-finite results which are numerically close to the result of the full process at tree level and at one-loop order, where an agreement of better than $1\,\%$ is found for the considered process. The most accurate prediction for this process based on the generalised NWA, taking into account also corrections that are formally of higher orders, is briefly discussed.

## Introduction

The description of the fundamental interactions of nature in terms of quantum field theories that are evaluated perturbatively has been extraordinarily successful in the context of elementary particle physics. Nevertheless, this theoretical formulation is plagued by a long-standing problem [1–3], since the asymptotic in- and outgoing states of quantum field theories are defined at infinite times corresponding to stable incoming and outgoing particles, while collider physics processes usually involve numerous unstable particles, see e.g. Refs. [4–7]. While in principle it would be possible to perform calculations of the theoretical predictions for the full process of stable incoming and outgoing particles, this is in many cases not feasible in practice (and still leaves the problem of the treatment of intermediate particles that can become resonant). Instead, one often seeks to simplify the task of calculating a more complicated process by separately treating the production of on-shell particles and their decays, where the latter can happen in several separate steps, each resulting in on-shell outgoing particles. Such an approach of simplifying the task of computing a complicated process involving many particles in the final state is in particular crucial in the context of incorporating higher-order corrections.

The separation of a more complicated process into several sub-processes involving on-shell particles as incoming and outgoing states is achieved with the help of the “narrow-width approximation” (NWA) for particles having a total width that is much smaller than their mass. The application of the NWA is beneficial since the sub-processes can often be calculated at a higher loop order than it would be the case for the full process, and it is also useful in terms of computational speed. Indeed, many Monte-Carlo generators make use of the NWA. An important condition limiting the applicability of this approximation, however, is the requirement that there should be no interference of the contribution of the intermediate particle for which the NWA is applied with any other close-by resonance, see e.g. Refs. [8–10]. While within the Standard Model (SM) of particle physics this condition is usually valid for relevant processes at high-energy colliders such as the LHC or a future Linear Collider, many models of physics beyond the SM (BSM) have mass spectra where two or more states can be nearly mass-degenerate. If the mass gap between two intermediate particles is smaller than the sum of their total widths, the interference term between the contributions from the two nearly mass-degenerate particles may become large.

For instance, mass degeneracies can be encountered in the Minimal Supersymmetric extension of the Standard Model (MSSM) [11–13]. In particular, the MSSM may contain approximately mass-degenerate first and second generation squarks and sleptons. In the decoupling limit [14], the MSSM predicts a SM-like light Higgs boson, which can be compatible with the signal discovered by ATLAS [15] and CMS [16] at a mass of about $M_h \simeq 125$ GeV, and two further neutral Higgs bosons and a charged Higgs boson $H^\pm$, which are significantly heavier and nearly mass-degenerate. While in the $\mathcal {CP}$-conserving case the heavy neutral Higgs bosons *H* and *A* are $\mathcal {CP}$-eigenstates and therefore do not mix with each other, $\mathcal {CP}$-violating loop contributions can induce sizable interference effects, see e.g. Ref. [10]. The compatibility of degenerate NMSSM Higgs masses with the observed Higgs decay rate into two photons was recently pointed out e.g. in Ref. [17]. Another example are degenerate Higgs bosons in (non-supersymmetric) two-Higgs doublet models, see e.g. Refs. [18, 19]. Furthermore, degeneracies can also occur in models of (universal) extra dimensions where the masses at one Kaluza–Klein level are degenerate up to their SM masses and loop corrections, see for example Refs. [20–22]. Small mass differences of sequential $Z'$ and $W'$ bosons are analysed in an extension of the SM as an effective field theory in Ref. [23]. On the other hand, models with new particles on various mass levels often exhibit long cascade decays, so that there is a particular need in these cases for an approximation with which the complicated full process can be simplified into smaller pieces that can be treated more easily. However, several cases have been identified in the literature in which the NWA is insufficient due to sizeable interference effects, e.g. in the context of the MSSM in Refs. [8, 9, 24–26] and in the context of two- and multiple-Higgs models and in Higgsless models in Ref. [27].

In the following we present a generalised NWA (gNWA), which extends the standard NWA (sNWA) by providing a factorisation into on-shell production and decay while taking into account interference effects. In Ref. [10] such a method was introduced at the tree level and applied to interference effects in the MSSM Higgs sector. This method was further extended in Ref. [28], in particular by incorporating partial loop contributions into an interference weight factor. A similar coupling-based estimation of an interference between new heavy quarks at lowest order was suggested in Ref. [29]. Interfering new, nearly degenerate vector bosons were considered in Ref. [23] in an approach of the product of involved couplings and on-shell parton luminosities. In the present paper we formulate a gNWA based on an on-shell evaluation of the interference contributions which is applicable at the loop level, incorporating factorisable virtual and real corrections. We validate the method for an example process by confronting the one-loop result within the gNWA with the result of the full process at the one-loop level. We furthermore investigate different levels of approximations, where we compare the on-shell matrix elements in the interference term with possible further simplifications based on interference weight factors. In the considered example process we study interference effects between the two neutral $\mathcal {CP}$-even MSSM Higgs bosons *h* and *H* in the decay of a heavy neutralino and the subsequent decay into a fermion pair. Besides the validation against the full result for this process we also discuss additional improvements by the incorporation of corrections that are formally of higher orders. The discussed cases are meant to illustrate that the proposed method is applicable to a wide range of possible processes in different models.

The paper is structured as follows. Section 2 reviews the standard NWA before introducing the interference-improved extension in two different versions in Sect. 3. The notation of the parts of the MSSM that are needed for the phenomenological discussion in the following sections is defined in Sect. 4, with particular emphasis on the mixing of Higgs bosons. In Sect. 5, the gNWA is applied at the tree level to the example process of Higgs production from the decay of a heavier neutralino and its subsequent decay into a pair of $\tau$-leptons. The numerical results for those contributions are discussed in Sect. 6. In Sect. 7 the application of the gNWA at the loop level is demonstrated. For comparison, the full one-loop calculation of the example process is performed in Sect. 8, including vertex, propagator, box and bremsstrahlung corrections. The numerical comparison and accordingly the validation of the gNWA at NLO is discussed in Sect. 9, where also the accuracy of the gNWA is investigated. Section 10 contains our conclusions.

## Standard narrow-width approximation

**Fig. 1:**
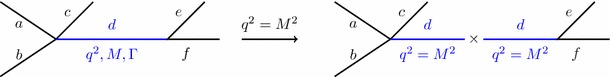
The resonant process $ab \rightarrow cef$ is split into the production $ab \rightarrow cd$ and decay $d\rightarrow ef$ with particle *d* on-shell

The narrow-width approximation is a useful way to simplify the calculation of complicated processes involving the resonant contribution of an unstable particle. The basic idea is to factorise the whole process into the on-shell production and the subsequent decay of the resonant particle. The following picture in Fig. 1 visualises this splitting using the example of an arbitrary process $ab \rightarrow cef$ with an intermediate particle *d*.

In the following, we focus on scalar propagators. Nonetheless, although the production and decay are calculated independently, the spin of an intermediate particle can be taken into account by means of spin correlations [30, 31] giving rise to spin–density matrices. While we do not consider the non-zero spin case explicitly, the formalism of spin–density matrices should be applicable to the gNWA discussed below in the same way as for the sNWA.

### Unstable particles and the total decay width

Since the total width $\Gamma$ plays a crucial role in resonant production and decay, we will briefly discuss resonances and unstable particles, see e.g. Refs. [32, 33]. While stable particles are associated with a real pole of the S-matrix, for unstable particles the associated self-energy develops an imaginary part, so that the pole of the propagator is located off the real axis within the complex plane. For a single pole $M_\mathrm{c}$, the scattering matrix as a function of the squared centre-of-mass energy *s* can be written in the vicinity of the complex pole in a gauge-invariant way as

1 $$\begin{aligned} \mathcal {M}(s) = \frac{R}{s-M_\mathrm{c}^{2}}+F(s) , \end{aligned}$$

where *R* denotes the residue and *F* represents non-resonant contributions. Writing the complex pole as $M_\mathrm{c}^{2}=M^{2}-iM\Gamma$, the mass *M* of an unstable particle is obtained from the real part of the complex pole, while the total width is obtained from the imaginary part. Accordingly, the expansion around the complex pole leads to a Breit–Wigner propagator with a constant width,

2 $$\begin{aligned} \Delta ^{\text {W}}(q^{2}):=\frac{1}{q^2 - M^2 + i M\Gamma } . \end{aligned}$$

In the following, we will use a Breit–Wigner propagator of this form to express the contribution of the unstable scalar *d* with mass *M* and total width $\Gamma$ in the resonance region (a Breit–Wigner propagator with a running width can be obtained from a simple reparametrisation of the mass and width appearing in Eq. (2)).

The NWA is based on the observation that the on-shell contribution in Eq. (2) is strongly enhanced if the total width is much smaller than the mass of the particle, $\Gamma \ll M$. Within its range of validity (see the discussion in the following section) the NWA provides an approximation of the cross section for the full process in terms of the product of the production cross section (or the previous step in a decay cascade) times the respective branching ratio:

3 $$\begin{aligned} \sigma _{ab \rightarrow cef} \simeq \sigma _{ab \rightarrow cd} \times \text {BR}_{d\rightarrow ef}. \end{aligned}$$

### Conditions for the narrow-width approximation

The NWA can only be expected to hold reliably if the following prerequisites are fulfilled (see e.g. Refs. [8, 34]):

- A narrow mass peak is required in order to justify the on-shell approximation. Otherwise off-shell effects may become large, cf. e.g. [24, 35, 36].
- Furthermore, the propagator needs to be separable from the matrix element. However, loop contributions involving a particle exchange between the initial and the final state give rise to non-factorisable corrections. Hence, the application of the NWA beyond lowest order relies on the assumption that the non-factorisable and non-resonant contributions are sufficiently suppressed compared to the dominant contribution where the unstable particle is on resonance. Concerning the incorporation of non-factorisable but resonant contributions from photon exchange, see e.g. Ref. [37].
- Both sub-processes have to be kinematically allowed. For the production of the intermediate particle, this means that the centre of mass energy $\sqrt{s}$ must be well above the production threshold of the intermediate particle with mass *M* and the other particles in the final state of the production process, i.e. $\sqrt{s}\gg M\,+\,m_\mathrm{c}$ for the process shown in Fig. 1. Otherwise, threshold effects must be considered [38].
- On the other hand, the decay channel must be kinematically open and sufficiently far above the decay threshold, i.e. $M \gg \sum m_{f}$, where $m_{f}$ are the masses of the particles in the final state of the decay process, here $m_{e}\,+\,m_{f}$. Off-shell effects can be enhanced if intermediate thresholds are present. This is the case for instance for the decay of a Higgs boson with a mass of about 125 GeV into four leptons. Since for an on-shell Higgs boson of this mass this process is far below the threshold for on-shell *WW* and *ZZ* production, it suffers from a significant phase-space suppression. Off-shell Higgs contributions above the threshold for on-shell *WW* and *ZZ* production are therefore numerically more important than one would expect just from a consideration of $\Gamma /M$ [39].
- As another crucial condition, interferences with other resonant or non-resonant diagrams have to be small because the mixed term would be neglected in the NWA. The major part of the following chapters is dedicated to a generalisation of the NWA for the inclusion of interference effects of nearly mass degenerate states, see also Refs. [10, 28].

### Factorisation of the phase space and cross section

In order to fix the notation used for the formulation of the gNWA in Sect. 3, we review some kinematic relations.

**The phase space** The phase space $\Phi$ is a Lorentz invariant quantity. Its differential is denoted as differential Lorentz invariant phase space (dlips) or $\mathrm{d}\Phi _n$. It is characterised by the number *n* of particles in the final state [40]

4 $$\begin{aligned} \mathrm{d}\Phi _n\equiv & {} \mathrm{dlips}\left( P;p_1,\ldots ,p_n\right) \nonumber \\= & {} (2\pi )^4\delta ^{(4)}\left( P-\sum \limits _{f=1}^np_f\right) \displaystyle \prod _{f=1}^n\frac{\mathrm{d}^3p_f}{(2\pi )^32E_f}. \end{aligned}$$

**Factorisation** Equation (3) is based on the property of the phase space and the matrix element to be factorisable into sub-processes. The phase space element $\mathrm{d}\Phi _n$ with *n* particles in the final state as in Eq. (4) will now be expressed as a product of the *k*-particle phase space $\Phi _k$ with $k < n$ and the remaining $\Phi _{n-k+1}$ [40, 41],

5 $$\begin{aligned} \mathrm{d}\Phi _n = \mathrm{d}\Phi _k \frac{\mathrm{d}q^2}{2\pi } \mathrm{d}\Phi _{n-k+1}, \end{aligned}$$

where *q* denotes the momentum of the resonant particle. Now $\Phi _k(q)$ can be interpreted as the *production* phase space $P \rightarrow \left\{ p_1, \ldots ,p_{k-1},q\right\}$ and $\Phi _{n-k+1}(q)$ as the *decay* phase space $q \rightarrow \left\{ p_k,\ldots ,p_n\right\}$. The factorisation of $\mathrm{d}\Phi _n$ is exact, no approximation has been made so far. Next, we rewrite the amplitude with a scalar propagator as a product of the production (P) and decay (D) part. Beyond the tree level, this is only possible if non-factorisable loop-contributions are absent or negligible,

6 $$\begin{aligned}&\!\!\!\mathcal {M} = \mathcal {M}_{P}\frac{1}{q^2 - M^2 +iM\Gamma }\mathcal {M}_{D}\nonumber \\&\!\!\!\quad \Rightarrow |\mathcal {M}|^2 = |\mathcal {M}_{P}|^{2}\frac{1}{(q^2 - M^2)^2 +(M\Gamma )^2}|\mathcal {M}_{D}|^{2}. \end{aligned}$$

One can distinguish two categories of processes. On the one hand, for a scattering process $a,b \rightarrow X$ to any final state *X* (in particular $a,b\rightarrow c,e,f$ for the example in Fig. 1), the flux factor is given by $F=2\lambda ^{1/2}(s,m_a^{2},m_b^{2})$ with the kinematic function [41]

7 $$\begin{aligned} \lambda (x,y,z) := x^{2}+y^{2}+z^{2}-2(xy+yz+zx). \end{aligned}$$

On the other hand, for a decay process $a\rightarrow X$ (for example $a\rightarrow c,e,f$), the flux factor is determined by the mass of the decaying particle, $F=2m_a$. Then the full cross section is given as (the sum/average over spin states where appropriate is implicitly understood)

8 $$\begin{aligned} \sigma = \frac{1}{F}\int \mathrm{d}\Phi |\mathcal {M}|^2 . \end{aligned}$$

For the decomposition into production and decay, we do not only factorise the matrix elements as in Eq. (6). Based on Eq. (5), also the phase space of the full process is factorised into the production phase space $\Phi _{P}$ and the decay phase space $\Phi _{D}$ (here defined for the example process in Fig. 1, but they can be generalised to other external momenta), which depend on the momentum of the resonant particle:

9 $$\begin{aligned}&\mathrm{d}\Phi = \mathrm{dlips}(\sqrt{s}; p_c, p_e, p_f)\nonumber \\&\mathrm{d}\Phi _{P}= \mathrm{dlips}(\sqrt{s}; p_c, q)\nonumber \\&\mathrm{d}\Phi _{D} = \mathrm{dlips}(q; p_e, p_f). \end{aligned}$$

Under the assumption of negligible non-factorisable loop contributions, one can then express the cross section in (8) as

10 $$\begin{aligned} \sigma= & {} \frac{1}{F}\int \frac{\mathrm{d}q^2}{2\pi }\left( \int \mathrm{d}\Phi _{P} |\mathcal {M}_{P}|^{2}\right) \nonumber \\&\times \frac{1}{(q^2 - M^2)^2 +(M\Gamma )^2}\left( \int \mathrm{d}\Phi _{D} |\mathcal {M}_{D}|^{2}\right) . \end{aligned}$$

In this analytical formula of the cross section, the production and decay matrix elements and the sub-phase spaces are separated from the Breit–Wigner propagator. However, the full $q^{2}$-dependence of the matrix elements and the phase space is retained. The off-shell production cross section of a scattering process with particles $a,\,b$ in the initial state and the production flux factor *F* reads

11 $$\begin{aligned} \sigma _{P}(q^{2}) =\frac{1}{F}\int \mathrm{d}\Phi _{P} |\mathcal {M}_{P}(q^{2})|^{2}. \end{aligned}$$

The decay rate of the unstable particle, $d\rightarrow ef$, with energy $\sqrt{q^{2}}$ is obtained from the integrated squared decay matrix element divided by the decay flux factor $F_{D}=2\sqrt{q^{2}}$,

12 $$\begin{aligned} \Gamma _{D}(q^{2}) = \frac{1}{F_{D}}\int \mathrm{d}\Phi _{D}|\mathcal {M}_{D}(q^{2})|^{2} . \end{aligned}$$

Hence one can rewrite the full cross section from Eq. (10) as

13 $$\begin{aligned} \sigma = \int \frac{\mathrm{d}q^{2}}{2\pi } \sigma _{P}(q^{2})\frac{2\sqrt{q^{2}}}{(q^2 - M^2)^2 +(M\Gamma )^2} \Gamma _{D}(q^{2}). \end{aligned}$$

In the limit where $(\Gamma M)\rightarrow 0$ the Dirac $\delta$-distribution emerges from the Cauchy distribution,

14 $$\begin{aligned} \lim \limits _{(M\Gamma )\rightarrow 0}\frac{1}{(q^{2}-M^{2})^2+(M\Gamma )^2} = \delta (q^{2}-M^{2})\frac{\pi }{M\Gamma }. \end{aligned}$$

For the integration of the $\delta$-distribution, the integral boundaries are shifted from $q^{2}_\mathrm{{max}},\,q^{2}_\mathrm{{min}}$, i.e. the upper and lower bound on $q^{2}$, respectively, to $\pm \infty$ because the contributions outside the narrow resonance region are expected to be small. So this extension of the integral should not considerably alter the result. The zero-width limit implies that the production cross section, decay width and the factor $\sqrt{q^{2}}$ are evaluated on-shell at $q^{2}=M^{2}$. This applies both to the matrix elements and the phase space elements. The described approximation leads to the well-known factorisation into the production cross section times the decay branching ratio,

15 $$\begin{aligned}&\sigma \mathop {\rightarrow }\limits ^{(M\Gamma )\rightarrow 0}\int \limits _{-\infty }^{+\infty } \frac{\mathrm{d}q^{2}}{2\pi }\sigma _{P}(q^{2})\,2\sqrt{q^{2}}\,\delta (q^{2}-M^{2})\frac{\pi }{M\Gamma }\,\Gamma _{D}(q^{2})\nonumber \\&\!\!\!\quad =\sigma _{P}(M^{2})\cdot \frac{\Gamma _{D}(M^{2})}{\Gamma } \equiv \sigma _{P}\cdot \text {BR}, \end{aligned}$$

with the branching ratio $\text {BR}=\Gamma _{D}/\Gamma$, where $\Gamma _{D}$ denotes the partial decay width into the particles in the final state of the considered process, and $\Gamma$ is the total decay width of the unstable particle. While Eq. (15) has been obtained in the limit $(M\Gamma )\rightarrow 0$, it is expected to approximate the result for non-zero $\Gamma$ up to terms of $\mathcal {O}(\frac{\Gamma }{M})$.

Going beyond the approximation of Eq. (15) for the treatment of finite width effects, the on-shell approximation can be applied just to the matrix elements for production and decay (if both subprocesses are kinematically allowed, as mentioned in Sect. 2.2) while keeping a finite width in the integration over the Breit–Wigner propagator in the form of Eq. (13). This is gauge invariant and motivated by the consideration that the Breit–Wigner function is rapidly falling causing that only matrix elements close to the mass shell $q^{2}=M^{2}$ contribute significantly. It results in a modified NWA improved for off-shell effects, see e.g. Refs. [39, 42],

16 $$\begin{aligned} \sigma ^{(\mathrm{ofs})}\!=\! \sigma _{P}(M^{2})\left[ \int \frac{\mathrm{d}q^{2}}{2\pi } \frac{2M}{(q^2 \!-\! M^2)^2 \!+\!(M\Gamma )^2} \right] \Gamma _{D}(M^{2}). \end{aligned}$$

## Formulation of a generalised narrow-width approximation

### Cross section with interference term

If all conditions in Sect. 2.2 are met, the NWA is expected to work reliably up to terms of $\mathcal {O}(\frac{\Gamma }{M})$. This section addresses the issue of how to extend the NWA such that interference effects can be included, leading to a generalised NWA [10, 28]. Interference effects can be large if there are several resonant diagrams whose intermediate particles are close in mass compared to their total decay widths:

17 $$\begin{aligned} |M_1 - M_2| \lesssim \Gamma _1+\Gamma _2. \end{aligned}$$

In these nearly mass-degenerate cases, the Breit–Wigner functions $\Delta ^{\text {BW}}_1(q^2),~ \Delta ^{\text {BW}}_2(q^2)$ overlap significantly, and an integral of the form

18 $$\begin{aligned} \int \limits _{q^2_\mathrm{{min}}}^{q^2_\mathrm{{max}}}\mathrm{d}q^{2}\Delta ^{\text {BW}}_1(q^2)\Delta ^{*\text {BW}}_2(q^2)\cdot f(\mathcal {M},p_i,\ldots ) \end{aligned}$$

is not negligible. The boundaries $q^{2}_\mathrm{{min}}, q^{2}_\mathrm{{max}}$ are the lower and upper limits of the kinematically allowed region of $q^{2}$, and *f* summarises a possible dependence on matrix elements $\mathcal {M}$ and momenta $p_i$ in the phase space. Such interference effects might especially be relevant in models of new physics where an enlarged particle spectrum allows for more possibilities of mass degeneracies in some parts of the parameter space.

Let $h_1, h_2$ be two resonant intermediate particles, for example two Higgs bosons, with similar masses occurring in a process $ab \rightarrow cef$, i.e. $ab \rightarrow c h_i, h_i \rightarrow ef$ (cf. Fig. 1 with $d=h_1,h_2$). If non-factorisable loop corrections can be neglected, the full matrix element (dropping the $q^{2}$-dependence of the matrix elements to simplify the notation) is given by (as mentioned above, see Sect. 2, we explicitly treat the case of scalar resonant particles; spin correlations of intermediate particles with non-zero spin can be taken into account using spin–density matrices)

19 $$\begin{aligned} \mathcal {M}&=\mathcal {M}_{ab\rightarrow c h_1}\frac{1}{q^2-M_1^2 + iM_1\Gamma _1}\mathcal {M}_{h_1\rightarrow ef} \nonumber \\&\quad + \mathcal {M}_{ab\rightarrow c h_2}\frac{1}{q^2-M_2^2 + iM_2\Gamma _2}\mathcal {M}_{h_2\rightarrow ef}. \end{aligned}$$

The squared matrix element contains the two separate contributions of $h_1,\,h_2$ and in the second line of Eq. (20) the interference term,

20 $$\begin{aligned} |\mathcal {M}|^2&=\frac{|\mathcal {M}_{ab\rightarrow c h_1}|^2|\mathcal {M}_{h_1\rightarrow ef}|^2}{(q^2 \!-\! M_1^2)^2 \!+\! M_1^2 \Gamma _1^2}+\frac{|\mathcal {M}_{ab\rightarrow c h_2}|^2|\mathcal {M}_{h_2\rightarrow ef}|^2}{(q^2 \!-\! M_2^2)^2 \!+\! M_2^2 \Gamma _2^2}\nonumber \\&\quad +\,2 \mathrm{Re}\left\{ \frac{\mathcal {M}_{ab\rightarrow c h_1}\mathcal {M}^*_{ab\rightarrow c h_2}\mathcal {M}_{ h_1\rightarrow ef}\mathcal {M}^*_{h_2\rightarrow ef}}{(q^2-M_1^2 + iM_1\Gamma _1)(q^2-M_2^2 - iM_2\Gamma _2)} \right\} . \end{aligned}$$

So the full cross section from Eq. (13) with the matrix element from Eq. (20) can be written as

21 $$\begin{aligned} \sigma _{ab\rightarrow cef}&=\int \frac{\mathrm{d}q^{2}}{2\pi }\left[ \frac{\sigma _{ab\rightarrow ch_1}(q^{2})~2\sqrt{q^{2}}~ \Gamma _{h_1\rightarrow ef}(q^{2})}{(q^2 - M_{h_1}^2)^2 +(M_{h_1}\Gamma _{h_1})^2}\right. \nonumber \\&\!\!\!\quad \left. +\, \frac{\sigma _{ab\rightarrow ch_2}(q^{2})~2\sqrt{q^{2}}~ \Gamma _{h_2\rightarrow ef}(q^{2})}{(q^2 - M_{h_2}^2)^2 +(M_{h_2}\Gamma _{h_2})^2}\right] \nonumber \\&\!\!\!\quad +\int \frac{\mathrm{dlips}(s;p_c,q)\mathrm{d}q^{2}\mathrm{dlips}(q;p_e,p_f)}{2\pi \cdot 2\lambda ^{1/2}(s,m_a^{2},m_b^{2})}\nonumber \\&\!\!\!\quad \times 2\mathrm{Re}\left\{ \frac{\mathcal {M}_{ab\rightarrow c h_1}\mathcal {M}^*_{ab\rightarrow c h_2}\mathcal {M}_{ h_1\rightarrow ef}\mathcal {M}^*_{h_2\rightarrow ef}}{(q^2-M_1^2 + iM_1\Gamma _1)(q^2-M_2^2 - iM_2\Gamma _2)}\right\} . \end{aligned}$$

We will use Eq. (21) as a starting point for approximations of the full cross section. The first two terms can again be approximated by the finite-narrow-width approximation according to Eq. (16), or by the usual narrow-width approximation in the limit of a vanishing width from Eq. (15) as $\sigma \times \text {BR}$. The interference term still consists of an integral over the $q^{2}$-dependent matrix elements, the product of Breit–Wigner propagators and the phase space.

### On-shell matrix elements

Our approach is to evaluate the production ($\mathcal {P}$) and decay ($\mathcal {D}$) matrix elements

22 $$\begin{aligned} \mathcal {P}_i(q^{2}) \equiv \mathcal {M}_{ab\rightarrow ch_i}(q^{2}), \quad \mathcal {D}_i(q^{2}) \equiv \mathcal {M}_{h_i\rightarrow ef}(q^{2}) \end{aligned}$$

on the mass shell of the intermediate particle $h_i$ [28]. This is motivated by the assumption of a narrow resonance region $[M_{h_i}-\Gamma _{h_i}, M_{h_i}+\Gamma _{h_i}]$ so that off-shell contributions of the matrix elements in the integral are suppressed by the non-resonant tail of the Breit–Wigner propagator if $\mathcal {P}$ and $\mathcal {D}$ vary only mildly^1^ with $q^{2}$. Then the interference term from the last line of Eq. (21) is approximated by

23 $$\begin{aligned} \sigma _\mathrm{{int}}&= \int \frac{\mathrm{d}\Phi _{P} \mathrm{d}q^{2} \mathrm{d}\Phi _{D}}{2\pi F}\nonumber \\&\quad \times 2\mathrm{Re}\frac{\mathcal {P}_{1}(q^{2})\mathcal {P}^*_{2}(q^{2})\mathcal {D}_{1}(q^{2})\mathcal {D}^*_{2}(q^{2})}{(q^2-M_1^2 + iM_1\Gamma _1)(q^2-M_2^2 - iM_2\Gamma _2)} \end{aligned}$$

24 $$\begin{aligned}&= \frac{2}{F}\mathrm{Re}\int \frac{\mathrm{d}q^{2}}{2\pi }\Delta ^{\text {BW}}_1(q^{2}) \Delta ^{*\text {BW}}_2(q^{2})\nonumber \\&\quad \times \left[ \int \mathrm{d}\Phi _{P}(q^{2})\mathcal {P}_{1}(q^{2})\mathcal {P}^*_{2}(q^{2})\right] \nonumber \\&\quad \times \,\left[ \int \mathrm{d}\Phi _{D}(q^{2}) \mathcal {D}_{1}(q^{2})\mathcal {D}^*_{2}(q^{2})\right] \nonumber \\&\simeq \frac{2}{F}\mathrm{Re}\int \frac{\mathrm{d}q^{2}}{2\pi }\Delta ^{\text {BW}}_1(q^{2}) \Delta ^{*\text {BW}}_2(q^{2})\nonumber \\&\quad \times \left[ \int \mathrm{d}\Phi _{P}(q^{2})\mathcal {P}_{1}(M_1^{2})\mathcal {P}^*_{2}(M_2^{2})\right] \nonumber \\&\quad \times \,\left[ \int \mathrm{d}\Phi _{D}(q^{2}) \mathcal {D}_{1}(M_1^{2})\mathcal {D}^*_{2}(M_2^{2}) \right] . \end{aligned}$$

Equation (24) represents our master formula for the interference contribution. At this stage, we have only evaluated the matrix elements on the mass shell of the particular Higgs boson by setting $q^{2}=M_{h_i}^{2}$ (this is also important for ensuring the gauge invariance of the considered contributions). So the on-shell matrix elements can be taken out of the $q^{2}$-integral. But the dependence of the matrix elements on further invariants and momenta is kept. For 2-body decays, it is possible to carry out the phase space integration without referring to the specific form of the matrix elements. In general, however, the matrix elements are functions of the phase space integration variables.

The approximation in Eq. (24) is a simplification of the full expression in Eq. (23) since the integrand of the $q^{2}$-integral is simplified. We will use Eq. (24) in the numerical calculation of an example process in Sect. 5.

We will furthermore investigate additional approximations of the integral structure in Eq. (24), which would simplify the application of the gNWA. This issue is discussed at the tree level in the following section.

### On-shell phase space and tree level interference weight factors

The following discussion, which focuses on the tree-level case, concerns a technical simplification of the master formula in Eq. (24). It will be numerically applied in Fig. 5 below and extended to the 1-loop level in Sect. 7. As a possible further simplification on top of the on-shell approximation for matrix elements, one can also evaluate the production and decay phase spaces on-shell. This is based on the same argument as for the on-shell evaluation of the matrix elements because off-shell phase space elements are multiplied with the non-resonant tail of Breit–Wigner functions. Now the $q^{2}$-independent matrix elements and phase space integrals can be taken out of the $q^{2}$-integral,

25 $$\begin{aligned} \sigma _\mathrm{{int}}\simeq & {} \frac{2}{F}\mathrm{Re}\left\{ \left[ \int \mathrm{d}\Phi _{P}\mathcal {P}_{1}(M_1^{2})\mathcal {P}^*_{2}(M_2^{2})\right] \right. \nonumber \\&\times \left. \left[ \int \mathrm{d}\Phi _{D} \mathcal {D}_{1}(M_1^{2})\mathcal {D}^*_{2}(M_2^{2})\right] \right. \nonumber \\&\times \left. \int \frac{\mathrm{d}q^{2}}{2\pi }\Delta ^{\text {BW}}_1(q^{2})\Delta ^{*\text {BW}}_2(q^{2})\right\} . \end{aligned}$$

The choice at which mass, $M_1$ or $M_2$, to evaluate the production and decay phase space regions is not unique. We thus introduce a weighting factor between the two possible processes, as an ansatz based on their production cross sections and branching ratios:

26 $$\begin{aligned} w_i := \frac{\sigma _{P_i}\,\text {BR}_i}{\sigma _{P_1}\,\text {BR}_1+\sigma _{P_2}\,\text {BR}_2}. \end{aligned}$$

Then we define the on-shell phase space regions as

27 $$\begin{aligned} \mathrm{d}\Phi _{P/D} := w_1\,\mathrm{d}\Phi _{P/D}(q^{2}=M_1^{2}) + w_2\,\mathrm{d}\Phi _{P/D}(q^{2}=M_2^{2}). \end{aligned}$$

In Eq. (25), a universal integral over the Breit–Wigner propagators emerges:

28 $$\begin{aligned} I:=\int \limits _{q^{2}_\mathrm{{min}}}^{q^{2}_\mathrm{{max}}} \frac{\mathrm{d}q^{2}}{2\pi }\Delta ^{\text {BW}}_1(q^2)\,\Delta ^{*\text {BW}}_2(q^2), \end{aligned}$$

which is analytically solvable,

29 $$\begin{aligned} I = \left[ \frac{\text {arctan}\left[ \frac{\Gamma _1 M_1}{M_1^2-q^{2}}\right] +\text {arctan}\left[ \frac{\Gamma _2 M_2}{M_2^2-q^{2}}\right] +\frac{i}{2}(\ln [\Gamma _1^2 M_1^2+(M_1^2-q^{2})^2]-\ln [\Gamma _2^2 M_2^2+(M_2^2-q^{2})^2])}{2\pi i(M_1^2-M_2^2-i(M_1 \Gamma _1 + M_2 \Gamma _2))}\right] _{q^{2}_\mathrm{{min}}}^{q^{2}_\mathrm{{max}}}. \end{aligned}$$

In the limit of equal masses and widths, $M=M_1=M_2$ and $\Gamma =\Gamma _1=\Gamma _2$, the product of Breit–Wigner propagators would become the absolute square, and the integral is reduced to

30 $$\begin{aligned} I(M,\Gamma )&= \int \limits _{q^{2}_\mathrm{{min}}}^{q^{2}_\mathrm{{max}}} \mathrm{d}q^{2}\frac{1}{(q^{2}-M^{2})^{2} + (M\Gamma )^{2}} \nonumber \\&=\left[ -\frac{1}{M\Gamma }\,\text {arctan}\left[ \frac{M^{2}-q^{2}}{M\Gamma }\right] \right] _{q^{2}_\mathrm{{min}}}^{q^{2}_\mathrm{{max}}}. \end{aligned}$$

This absolute square of the Breit–Wigner function is also present in the usual NWA in Eq. (14), and for vanishing $\Gamma$ it can be approximated by a $\delta$-distribution. Here, however, we allow for different masses and widths from the two resonant propagators. We evaluate only the matrix elements and differential phase space on-shell, but we do not perform a zero-width approximation. This approach is analogous to the finite-narrow-width approximation without the interference term in Eq. (16).

Under the additional assumption of equal masses, the interference part can be approximated in terms of cross sections, branching ratios and couplings in order to avoid the explicit calculation of the product of unsquared amplitudes and their conjugates. This will also avoid the phase space integrals in the interference term as in Eq. (25).

For this purpose, each matrix element is written as the coupling of the particular production or decay process, $C_{P_i}$ or $C_{D_i}$, times the helicity part $p(M_i^{2})$ or $d(M_i^{2})$, respectively,

31 $$\begin{aligned} \mathcal {P}_{i}(M_i^{2}) = C_{P_i}\,p(M_i^{2}),\quad \mathcal {D}_{i}(M_i^{2}) = C_{D_i}\, \mathrm{d}(M_i^{2}). \end{aligned}$$

The on-shell calculation of helicity matrix elements is demonstrated in Sect. 5.3 where also left- and right-handed couplings are distinguished. Here we use the schematic notation of Eq. (31), but it could directly be replaced by the L/R-sum as in Eq. (75) below.

If we then make the additional assumption $M_1\simeq M_2$, the helicity matrix elements coincide, $p(M_1^{2})\simeq p(M_2^{2})$, $\mathrm{d}(M_1^{2})\simeq \mathrm{d}(M_2^{2})$, thus the matrix elements differ just by fractions of their couplings,

32 $$\begin{aligned} \mathcal {P}_{2}(M_2^{2}) \simeq \frac{C_{P_2}}{C_{P_1}}\mathcal {P}_{1}(M_1^{2}),\quad \mathcal {D}_{2}(M_2^{2}) \simeq \frac{C_{D_2}}{C_{D_1}}\mathcal {D}_{1}(M_1^{2}). \end{aligned}$$

This enables us to replace the products of an amplitude involving the resonant particle 1 with a conjugate amplitude of resonant particle 2 by absolute squares of amplitudes as follows, where $i,j \in \{1,2\}$, $i\ne j$, and no summation over indices is implied:

33 $$\begin{aligned} \sigma _\mathrm{{int}}&\mathop {\simeq }\limits ^{(25)} 2\mathrm{Re}\left\{ \left[ \frac{1}{F}\int \mathrm{d}\Phi _{P}\mathcal {P}_{1}\mathcal {P}^*_{2}\right] \left[ \frac{1}{2M_i} \int \mathrm{d}\Phi _{D} \mathcal {D}_{1}\mathcal {D}^*_{2}\right] \right. \nonumber \\&\qquad \,\times \, \left. 2M_i \int \frac{\mathrm{d}q^{2}}{2\pi }\Delta ^{\text {BW}}_1(q^{2})\Delta ^{*\text {BW}}_2(q^{2})\right\} \end{aligned}$$

34 $$\begin{aligned}&\mathop {\simeq }\limits ^{(31)} 2\mathrm{Re}\left\{ \left[ \frac{1}{F}\int \mathrm{d}\Phi _{P}|\mathcal {P}_{i}|^{2}\frac{C_{P_j}^{*}}{C_{P_i}^{*}}\right] \left[ \!\frac{1}{2M_i}\int \mathrm{d}\Phi _{D}|\mathcal {D}_{i}|^{2}\frac{C_{D_j}^{*}}{C_{D_i}^{*}}\right] \right. \nonumber \\&\qquad \,\left. \times \, 2M_i\int \frac{\mathrm{d}q^{2}}{2\pi }\Delta ^{\text {BW}}_1(q^{2})\Delta ^{*\text {BW}}_2(q^{2})\right\} \end{aligned}$$

35 $$\begin{aligned}&\mathop {=}\limits ^{(11, 12)} \sigma _{P_i}\, \Gamma _{D_i}\cdot 2M_i \cdot 2\mathrm{Re}\nonumber \\&\quad \quad \,\times \,\left\{ \frac{C_{P_j}^{*}C_{D_j}^{*}}{C_{P_i}^{*}C_{D_i}^{*}}\,\int \frac{\mathrm{d}q^{2}}{2\pi }\Delta ^{\text {BW}}_1(q^{2})\Delta ^{*\text {BW}}_2(q^{2}) \right\} \end{aligned}$$

36 $$\begin{aligned}&\quad =\sigma _{P_i}\, \text {BR}_i\cdot 2M_i\Gamma _i \cdot 2\mathrm{Re}\left\{ x_i\cdot I\right\} . \end{aligned}$$

In the last step, we divided and multiplied by the total width $\Gamma _i$ to obtain the branching ratio $\text {BR}_i = \frac{\Gamma _{D_i}}{\Gamma _i}$. The universal integral *I* over the overlapping Breit–Wigner propagators is given in Eq. (28). Furthermore, we defined a scaling factor as the ratio of couplings [10, 28, 29],

37 $$\begin{aligned} x_i := \frac{C_{P_j}^{*}C_{D_j}^{*}}{C_{P_i}^{*}C_{D_i}^{*}} = \frac{C_{P_i}C_{P_j}^{*}C_{D_i}C_{D_j}^{*}}{|C_{P_i}|^{2}|C_{D_i}|^{2}}. \end{aligned}$$

Using Eq. (36) and the scaling factor $x_i$ with $i=1,~ j=2$ or vice versa allows to express $\sigma _\mathrm{{int}}$ alternatively in terms of the cross section, branching ratio, mass and width of either of the resonant particle 1 or 2. Since no summation over *i* or *j* is implied in Eq. (36), both contributions are accounted for by the weighting factor $w_i\in [0,1]$ from Eq. (26).

Next, we summarise the components of $\sigma _\mathrm{{int}}$ apart from $\sigma _{P_i}$ and $\text {BR}_i$, which also occur in the usual NWA, in an interference weight factor

38 $$\begin{aligned} R_i&:= 2M_i \Gamma _i w_i\cdot 2\mathrm{Re}\left\{ x_i I \right\} . \end{aligned}$$

Hence, in this approximation of on-shell matrix elements and production and decay phase spaces with the additional condition of equal masses, the interference contribution can be written as the weighted sum

39 $$\begin{aligned} \sigma _\mathrm{{int}} \simeq \sigma _{P_1}\, \text {BR}_1\cdot R_1 + \sigma _{P_2}\, \text {BR}_2\cdot R_2 , \end{aligned}$$

or in terms of only one of the resonant particles,

40 $$\begin{aligned} \sigma _\mathrm{{int}}&\simeq \sigma _{P_i}\, \text {BR}_i\cdot 2\tilde{R}_i ,\end{aligned}$$

41 $$\begin{aligned} \tilde{R}_i&:= 2M_i \Gamma _i \cdot \mathrm{Re}\left\{ x_i I \right\} \equiv \frac{R_i}{2w_i} . \end{aligned}$$

Finally, we are able to express the cross section of the complete process in this *R*-factor approximation, comprising the exchange of the resonant particles 1 and 2 as well as their interference, in the following compact form

42 $$\begin{aligned} \sigma&\simeq \sigma _{P_1}\, \text {BR}_1\cdot (1+R_1) + \sigma _{P_2}\, \text {BR}_2\cdot (1+R_2) \end{aligned}$$

43 $$\begin{aligned}&\simeq \sigma _{P_i}\, \text {BR}_i\cdot \,(1+2\tilde{R}_i) + \sigma _{P_j}\, \text {BR}_j \end{aligned}$$

Furthermore, it is possible to replace the term $\sigma _i\,\text {BR}_i$ in the two separate processes without the interference term by the finite-width integral from Eq. (16).

### Discussion of the steps of approximations

In the previous sections, we presented two levels of approximations for the interference term with two resonant particles. The first approximation in Sect. 3.2 represents our main result. It relies only on the on-shell evaluation of the matrix elements, justified by a narrow resonance region, but no further assumptions (beyond those already used in the sNWA) are implied. Different masses and finite widths are taken into account. This version requires the explicit calculation of unsquared on-shell amplitudes, preventing the use of e.g. convenient spinor trace rules. Furthermore, the phase space integration depends on $q^{2}$ so that the universal, process-independent Breit–Wigner integral *I* from Eq. (28) does not appear here.

The second approximation in Sect. 3.3 has been formulated only at tree level so far. It is based on the additional approximation, motivated by the same argument as for the matrix elements, of setting the differential Lorentz invariant phase spaces on-shell at either mass, scaled by a weighting factor. This makes the $q^{2}$-integration easier because only the universal integral *I* is left. Furthermore, it avoids the unusual calculation of on-shell amplitudes in an explicit representation by expressing the interference part as an interference weight factor *R* in terms of cross sections, branching ratios, masses and widths, which are already needed in the simple NWA, plus the universal integral *I* and a scaling factor *x* which consists of the process-specific couplings. Yet, this approximation holds only for equal masses. As discussed in the context of Eq. (18), the interference term is largest if the Breit–Wigner shapes overlap significantly due to the relation $\Delta M \lesssim \Gamma _1 +\Gamma _2$. Nevertheless, the masses are not necessarily equal in the interference region. Instead, the overlap criterion in Eq. (17) can as well be satisfied if one of the widths is relatively large. In this respect, the equal-mass condition is more restrictive than the overlap criterion. However, the equal-mass constraint is just applied on the matrix elements and phase space, whereas different masses and widths are distinguished in the Breit–Wigner integral. The *R*-factor method is technically easier to handle because the constituents of *R* can be obtained by standard routines in the program packages such as FormCalc [43–47] and FeynHiggs [48–51] that we use in the numerical computation. For one example process, this is done in Sect. 5. An extension of the generalised narrow-width approximation to the 1-loop level is discussed in Sect. 7.

## Particle content and mixing in the MSSM

Before we discuss the application of the gNWA to an example process within the MSSM, we briefly summarise here the different particle sectors of the MSSM in order to clarify the notation and conventions.

### Propagator mixing in the Higgs sector

**Higgs sector at tree level** The MSSM contains two Higgs doublets,

44 $$\begin{aligned}&\mathcal {H}_1 = \begin{pmatrix} H_{11}\\ H_{12}\end{pmatrix} =\begin{pmatrix} v_1+\frac{1}{\sqrt{2}}(\phi _1^{0}-i\chi _1^{0})\\ -\phi _1^{-}\end{pmatrix}, \nonumber \\&\mathcal {H}_2 = \begin{pmatrix} H_{21}\\ H_{22}\end{pmatrix} = \begin{pmatrix} \phi _2^{+}\\ v_2+\frac{1}{\sqrt{2}}(\phi _2^{0}+i\chi _2^{0})\end{pmatrix} , \end{aligned}$$

with the vacuum expectation values $v_1, v_2$, respectively, whose ratio $\tan \beta \equiv \frac{v_2}{v_1}$ determines together with $M_{H^{\pm }}$ (or $M_A$) the MSSM Higgs sector at tree level. In principle, complex parameters can enter through loops, but in this methodical study we consider the MSSM with real parameters. The neutral fields $\phi _1^{0},\phi _2^{0},\chi _1^{0},\chi _2^{0}$ are rotated into the mass eigenstates *h*, *H*, *A*, *G*, where *h* and *H* are neutral $\mathcal {CP}$-even Higgs bosons (rotated from $\phi _1^{0},\phi _2^{0}$ by the mixing angle $\alpha$), *A* is the neutral $\mathcal {CP}$-odd Higgs boson and *G* denotes the neutral Goldstone boson. Besides, there are the charged Higgs and Goldstone bosons, $H^{\pm }, G^{\pm }$.

**Mixing in the MSSM Higgs sector** Higher-order corrections have a crucial impact on the phenomenology in the Higgs sector. We adopt the renormalisation scheme in the Higgs sector from Refs. [52, 53], where $M_A$ (or $M_{H^{\pm }}$) is renormalised on-shell while a $\overline{\text {DR}}$-renormalisation is used for the Higgs fields and $\tan \beta$. For the prediction of the considered process we incorporate important higher-order corrections from the Higgs sector already at the Born level by using for the Higgs-boson masses and total decay widths the predictions from FeynHiggs [48–51], which contain the full one-loop and dominant two-loop contributions.

Furthermore, because of the presence of off-diagonal self-energies like $\hat{\Sigma }_{ij}$ with $i,j=h, H, A$, the propagators of the neutral Higgs bosons mix with each other and in general also with contributions from the gauge and Goldstone bosons, see e.g. Refs. [52, 53]. The Higgs-boson masses therefore have to be determined from the complex poles of the Higgs propagator matrix.

For correct on-shell properties of external Higgs bosons the residues of the propagators have to be normalised to one. This is achieved by finite wave function normalisation factors, which can be collected in a matrix $\hat{\mathbf{Z }}$, such that for a one-particle irreducible vertex $\hat{\Gamma }_i$ with an external Higgs boson *i* the effect of Higgs mixing amounts to

45 $$\begin{aligned} \hat{\Gamma }_i \rightarrow \hat{\mathbf{Z }}_{ih}\hat{\Gamma }_h +\hat{\mathbf{Z }}_{iH}\hat{\Gamma }_H +\hat{\mathbf{Z }}_{iA}\hat{\Gamma }_A + \cdots , \end{aligned}$$

where the ellipsis indicates the mixing with the Goldstone and *Z*-bosons, which are not comprised in the $\hat{\mathbf{Z }}$-factors, but have to be calculated explicitly. The (non-unitary) matrix $\hat{\mathbf{Z }}$ can be written as (see Ref. [53])

46 $$\begin{aligned} \hat{\mathbf{Z }}&= \begin{pmatrix} \sqrt{\hat{Z}_h} &{}\quad \sqrt{\hat{Z}_h}\hat{Z}_{hH} &{}\quad \sqrt{\hat{Z}_h}\hat{Z}_{hA}\\ \sqrt{\hat{Z}_H}\hat{Z}_{Hh} &{}\quad \sqrt{\hat{Z}_H} &{}\quad \sqrt{\hat{Z}_H}\hat{Z}_{HA}\\ \sqrt{\hat{Z}_A}\hat{Z}_{Ah} &{}\quad \sqrt{\hat{Z}_A}\hat{Z}_{AH} &{}\quad \sqrt{\hat{Z}_A} \end{pmatrix}, \end{aligned}$$

with

47 $$\begin{aligned} \hat{Z}_{i} = \frac{1}{\frac{\partial }{\partial p^{2}}\frac{i}{\Delta _{ii}}}\bigg \vert _{p^{2}=M_{c_a}^{2}},\quad \hat{Z}_{ij} = \frac{\Delta _{ij}(p^{2})}{\Delta _{ii}(p^{2})}\bigg |_{p^{2}=M_{c_a}^{2}}, \end{aligned}$$

where the wave function normalisation factors are evaluated at the complex poles

48 $$\begin{aligned} M_{c_a}^{2} = M_{h_a}^{2} - i M_{h_a}\Gamma _{h_a} \end{aligned}$$

for $a=1,2,3$. We choose $a=1$ for $i=h$, $a=2$ for $i=H$ and $a=3$ for $i=A$. $M_{h_a}$ is the loop-corrected mass and $\Gamma _{h_a}$ the total width of $h_a$. In the $\mathcal {CP}$-conserving case, the $\hat{\mathbf{Z }}$-matrix is reduced to the $2\times 2$ mixing between *h* and *H*.

An amplitude involving resonant Higgs-boson propagators therefore needs to incorporate in general the full loop-corrected propagator matrix (and also the mixing contributions with the gauge and Goldstone bosons). It can be shown [10, 54] that in the vicinity of the resonance the full propagator matrix contribution can be approximated by

49 $$\begin{aligned} \sum _{i,j}\hat{\Gamma }_{i}^{\text {A}}\Delta _{ij}(p^{2})\hat{\Gamma }_{j}^{\text {B}} \simeq \sum _{\alpha ,i,j}\hat{\Gamma }_{i}^{\text {A}}\hat{\mathbf{Z }}_{\alpha i}\Delta ^\mathrm{{BW}}_{\alpha }(p^{2}) \hat{\mathbf{Z }}_{\alpha j}\hat{\Gamma }_{j}^{\text {B}}, \end{aligned}$$

involving the Breit–Wigner propagator $\Delta _{\alpha }^{\text {BW}}(p^{2})$ as given in Eq. (2), where $\hat{\Gamma }_{i}^{A}, \hat{\Gamma }_{j}^{B}$ are the one-particle irreducible vertices *A*, *B* of the Higgs bosons $i,\,j$, and $i,j,\alpha = h,H,A$ are summed over.

### The neutralino and chargino sector

The superpartners of the neutral gauge and Higgs bosons mix into the four neutralinos $\tilde{\chi }_i^{0}$, $i=1,2,3,4$, as mass eigenstates whose mass matrix is determined by the bino, wino and higgsino mass parameters $M_1, M_2, \mu$ and the parameter $\tan \beta$,

50 $$\begin{aligned} M_{\tilde{\chi }^{0}}&= \left( \begin{matrix} M_1 &{}\quad 0 &{}\quad -M_Z c_{\beta }s_W &{}\quad M_Z {{s}_{\beta }}s_W\\ 0 &{}\quad M_2 &{}\quad M_Z c_{\beta }c_W &{}\quad -M_Z {{s}_{\beta }}c_W \\ -M_Z c_{\beta }s_W &{}\quad M_Z c_{\beta }c_W &{}\quad 0 &{}\quad -\mu \\ M_Z {{s}_{\beta }}s_W &{}\quad -M_Z {{s}_{\beta }}c_W &{}\quad -\mu &{}\quad 0\\ \end{matrix} \right) . \end{aligned}$$

The admixture of gauginos and higgsinos in each neutralino can be determined from the components of the matrix *N* which diagonalises $M_{\tilde{\chi }^{0}}$ by $N^{*}M_{\tilde{\chi }^{0}}N^{-1}$.

The charginos $\tilde{\chi }_i^{\pm },~i=1,2$, as mass eigenstates are superpositions of the charged wino and higgsino,

51 $$\begin{aligned} M_{\tilde{\chi }^{\pm }} = \begin{pmatrix} M_2 &{}\quad \sqrt{2}M_W{{s}_{\beta }}\\ \sqrt{2}M_W c_{\beta }&{}\quad \mu \end{pmatrix}. \end{aligned}$$

The chosen example process requires the couplings at the Higgs–neutralino–neutralino and the Higgs–fermion–fermion vertices. For the neutralinos $\tilde{\chi }_i^{0},\tilde{\chi }_j^{0}$ with $i,j=1,2,3,4$ and the neutral Higgs bosons $h_{k}=\{h,H,A,G\}$ for $k=1,2,3,4$, the right-handed $C^{R}$ and left-handed $C^{L}$ neutralino-Higgs couplings are given by

52 $$\begin{aligned} C_{R}^{ijk}&=-C_{L*}^{ijk} = \frac{ie}{2c_{W}\,s_{W}}c^{ijk}, ~\text {with}\nonumber \\ c^{ijk}&= \left\{ \begin{matrix} (-s_{\alpha }N_{i3} -c_{\alpha }N_{i4})(s_{W}N_{j1}-c_{W}N_{j2}) \\ +\, (i\leftrightarrow j),~&{}k&{}=1,\\ (+c_{\alpha }N_{i3} -s_{\alpha }N_{i4})(s_{W}N_{j1}-c_{W}N_{j2})\\ +\, (i\leftrightarrow j),~&{}k&{}=2,\\ (+i{{s}_{\beta }}N_{i3} -ic_{\beta }N_{i4})(s_{W}N_{j1}-c_{W}N_{j2})\\ +\, (i\leftrightarrow j),~&{}k&{}=3,\\ (-ic_{\beta }N_{i3} -i{{s}_{\beta }}N_{i4})(s_{W}N_{j1}-c_{W}N_{j2})\\ +\, (i\leftrightarrow j),~&{}k&{}=4, \end{matrix} \right. \nonumber \\ \end{aligned}$$

where $s_{\alpha }\equiv \sin \alpha , c_{\alpha }\equiv \cos \alpha$ and likewise for $\beta$. With the left-/right-handed projection operators $\omega _{R/L}\equiv \frac{1}{2}(1\pm \gamma ^{5})$, the 3-point function of the neutralino-Higgs vertex is at tree level composed of

53 $$\begin{aligned} \Gamma ^{\text {tree}}_{\tilde{\chi }_{1}^{0},\chi _j,h_k} = {{\omega }_{R}}C^{ijk}_{R} \pm \omega _{L}C^{ijk}_{L}, \end{aligned}$$

where the + applies to the $\mathcal {CP}$-even Higgs bosons *h* and H, whilst the $-$ appears for the $\mathcal {CP}$-odd Higgs boson *A* and the Goldstone boson *G*. Mixing of Higgs bosons is taken into account by a linear combination of the couplings and Z-factors (see Sect. 4.1). In the following, the couplings $C_{h_kXY}$ always mean the mixed couplings (for $k,l,m = 1,2,3$; no summation over indices implied),

54 $$\begin{aligned} C_{h_kXY} \rightarrow \hat{\mathbf{Z }}_{h_k h_k} C_{h_kXY} + \hat{\mathbf{Z }}_{h_k h_l} C_{h_lXY} + \hat{\mathbf{Z }}_{h_k h_m} C_{h_mXY}.\quad \end{aligned}$$

For the calculation of higher-order corrections to the neutralino-chargino sector, it is essential to identify a stable renormalisation scheme according to the gaugino parameter hierarchy of $M_1, M_2$ and $\mu$ as it was pointed out in Refs. [10, 55, 56]. Choosing the external neutralinos and charginos in the considered process to be on-shell does not necessarily lead to the most stable renormalisation scheme. Among the four neutralinos and two charginos, three can be renormalised on-shell in relation to the three gaugino parameters $M_1, M_2$ and $\mu$. The most bino-, wino- and higgsino-like states should be chosen on-shell so that the three parameters are sufficiently constrained by the renormalisation conditions. Otherwise, an unstable choice of the input states can lead to unphysically large values for the parameter counterterms and mass corrections. On-shell renormalisation conditions were derived in Refs. [55, 57–63] for the MSSM with real parameters and in Refs. [56, 64, 65] for the complex case. Schemes with two charginos and one neutralino on-shell are referred to as NCC, with one chargino and two neutralinos as NNC and with three neutralinos input states as NNN. In view of our example process (see Sect. 5) and the gaugino hierarchy of the scenario, we will comment on our choice of a renormalisation scheme in Sect. (8.1.1).

### The sfermion and the gluino sectors

The mixing of sfermions $\tilde{f}_L, \tilde{f}_R$ within one generation into mass eigenstates $\tilde{f}_1, \tilde{f}_2$ is parametrised by the matrix

55 $$\begin{aligned} M_{\tilde{f}}&= \left( \begin{array}{cc} M_{\tilde{f}_L}^{2}\!+\!m_f^{2}\!+\!M_Z^{2}\cos 2 \beta (I_3^{f}\!-\!Q_fs_{W}^{2}) &{} m_f X_f^{*}\\ m_f X_f &{} M_{\tilde{f}_R}^{2}\!+\!m_{f}^{2}\!+\!M_Z^{2}\cos 2\beta Q_fs_{W}^{2} \end{array} \right) ,\end{aligned}$$

56 $$\begin{aligned} X_f&:= A_f - \mu ^{*}\cdot \left\{ \begin{matrix} \cot \beta ,~f = \text {up-type}~~~~\\ \tan \beta ,~f = \text {down-type}. \end{matrix} \right. \end{aligned}$$

The trilinear couplings $A_f$ as well as $\mu$ can be complex. Their phases enter the Higgs sector via sfermion loops, but as mentioned above here we only take real parameters into account. The sfermion masses at tree level are the eigenvalues of $M_{\tilde{f}}$. In the considered example process with *h*, *H* decaying into $\tau ^{+}\tau ^{-}$, the couplings of Higgs bosons to $\tau$-leptons are involved,

57 $$\begin{aligned} C_{h\tau \tau }^\mathrm{{tree}} = +\frac{\mathrm{igm}_{\tau }s_{\alpha }}{2M_W c_{\beta }},\quad C_{H\tau \tau }^\mathrm{{tree}} = -\frac{\mathrm{igm}_{\tau }c_{\alpha }}{2M_W c_{\beta }}. \end{aligned}$$

The mass of the gluino $\tilde{g}$ is given by $|M_3|$.

## Generalised narrow-width approximation at leading order: example process $\tilde{\chi }_4^0\rightarrow \tilde{\chi }_{1}^{0}\,h/H\rightarrow \tilde{\chi }_{1}^{0}\,\tau ^{+}\tau ^{-}$

The gNWA will be validated for a simple example process. The focus lies on providing a test case for the method rather than on the phenomenology of the process itself. For a comparison with the gNWA, we choose a process which can be calculated also at the 1-loop level without the on-shell approximation.

**Fig. 2:**
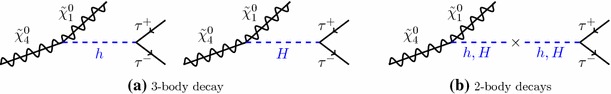
$\tilde{\chi }_4^0\rightarrow \tilde{\chi }_{1}^{0}\tau ^+ \tau ^-$ with *h* or *H* as intermediate particle in the two interfering diagrams. The decay process is either considered as **a** one 3-body decay or **b** decomposed in two 2-body decays

In the following, we will consider Higgs production from the decay of the heaviest neutralino and its subsequent decay into a pair of $\tau$-leptons, $\tilde{\chi }_4^0\rightarrow \tilde{\chi }_{1}^{0}\,h/H\rightarrow \tilde{\chi }_{1}^{0}\,\tau ^{+}\tau ^{-}$, which is a useful example process because it is computable as a full 3-body decay and it can be decomposed into two simple 2-body decays, see Fig. 2.

Moreover, the intermediate particles are scalars. Thus, for this process the treatment of interference effects can be trivially disentangled from any spin correlations between production and decay. Due to the neutralinos in the initial state and in the first decay step, soft bremsstrahlung only appears in the final state, and there is no photon exchange between the initial and final state. Restricting this test case to the MSSM with real parameters, only the two $\mathcal {CP}$-even states *h*, *H* mix due to $\mathcal {CP}$-conservation, instead of the $3\times 3$ mixing of *h*, *H*, *A* in the complex case. We neglect non-resonant diagrams from sleptons, which is a good approximation for the case of heavy sleptons. Slepton contributions to neutralino 3-body decays have been analysed in Ref. [63]. As a first step, we also neglect the exchange of an intermediate pseudoscalar *A*, Goldstone boson *G* and *Z*-boson for the purpose of a pure comparison of the factorised and the full Higgs contribution. For the most accurate prediction within the gNWA, which will be discussed in Sect. 9.4, we will add the tree-level *A*, *G*- and *Z*-exchange, but they do not interfere with *h* and *H* in the case of real parameters.

The decay width will be calculated using FeynArts [66–70] and FormCalc [43–47]^2^ both as a 3-body decay with the full matrix element and in the narrow-width approximation as a combination of two 2-body decays – with and without the interference term. In this and the following section, the gNWA will be applied at the tree level. The application at the loop level will follow conceptually in Sect. 8 and numerically in Sect. 9.

### 3-body decays: leading order matrix element

In order to compare the gNWA to the unfactorised LO result, we calculate the amplitude $\mathcal {M}_{h_k}$ of the 3-body decay via $h_k=h,H$. From the matrix element of the form

58 $$\begin{aligned} \mathcal {M}_{h_k}&= i C_{h_k \tilde{\chi }_i^{0}\tilde{\chi }_j^{0}}C_{h_k \tau \tau } \bar{u}(p_4,s_4)v(p_3, s_3) \nonumber \\&\quad \times \frac{1}{q^2 - M_{h_k}^2 + iM_{h_k}\Gamma _{h_k}}\bar{u}(p_2,s_2)u(p_1,s_1) \end{aligned}$$

we obtain the spin-averaged, squared amplitude consisting of the separate *h*, *H* contributions and the interference contribution,

59 $$\begin{aligned} \overline{|\mathcal {M}|^2}&=(p_1\cdot p_2 + m_{\tilde{\chi }_1^0}m_{\tilde{\chi }_4^0})(p_3\cdot p_4-m^2_{\tau })\nonumber \\&\quad \times \left( \frac{|C_{h \tilde{\chi }_{1}^{0}\tilde{\chi }_4^0}|^2|C_{h \tau \tau }|^2}{(q^2-m_h^2)^2 + m_h^2\Gamma _h^2} +\frac{|C_{H \tilde{\chi }_{1}^{0}\tilde{\chi }_4^0}|^2 |C_{H \tau \tau }|^2}{(q^2-m_H^2)^2 + m_H^2\Gamma _H^2}\right. \nonumber \\&\quad +\left. 2 \mathrm{Re}\left[ C_{h \tilde{\chi }_{1}^{0}\tilde{\chi }_4^0}C^*_{H \tilde{\chi }_{1}^{0}\tilde{\chi }_4^0}C_{h \tau \tau }C^*_{H \tau \tau } \!\cdot \! \Delta _h^{\text {BW}}(q^2) \Delta _H^{*\text {BW}}(q^2) \right] \right) \!, \end{aligned}$$

where the momenta and masses are labelled as $p_1\rightarrow p_2,p_3,p_4$ with $m_1 \equiv m_{\tilde{\chi }_4^0}, m_2\equiv m_{\tilde{\chi }_1^0}, m_3=m_4\equiv m_{\tau }$. In order to calculate the decay width in one of the Gottfried-Jackson frames [30], the products of momenta are rewritten in terms of two combined invariant masses, here e.g. $m_{23},m_{24}$:

60 $$\begin{aligned} p_1\cdot p_2&=\frac{1}{2}(m_{23}^2+m_{24}^2)-m_{\tau }^{2},\nonumber \\ p_3\cdot p_4&=\frac{1}{2}(m_1^2 + m_2^2 -m_{23}^2 - m_{24}^2)\nonumber ,\\ q^2&= (p_1-p_2)^2 = m_1^2 + m_2^2 -m_{23}^2-m_{24}^2. \end{aligned}$$

This yields the partial decay width for the 3-body decay [40],

61 $$\begin{aligned} \Gamma = \frac{1}{(2\pi )^3}\frac{1}{32m_{\tilde{\chi }_4^0}^3}\int |\mathcal {M}|^2\mathrm{d}m_{23}^{2}\mathrm{d}m_{24}^{2} \end{aligned}$$

which we will use for a comparison with the gNWA.

### Decomposition into 2-body decays

In this section, we calculate the 2-body decay widths of the subprocesses needed in the NWA. The matrix element for the production of $h_k=h,H$ is

62 $$\begin{aligned}&\mathcal {M}_{\tilde{\chi }_4^0\tilde{\chi }_{1}^{0}h_k} = i\bar{u}_2C_{h_k\tilde{\chi }_4^0\tilde{\chi }_{1}^{0}}u_1,\end{aligned}$$

63 $$\begin{aligned}&\overline{|\mathcal {M}_{\tilde{\chi }_4^0\tilde{\chi }_{1}^{0}h_k}|^2} = |C_{h_k\tilde{\chi }_4^0\tilde{\chi }_{1}^{0}}|^2 \,2\,(p_1\cdot p_2 +m_{\tilde{\chi }_4^0}m_{\tilde{\chi }_1^0}). \end{aligned}$$

In the rest frame of $\tilde{\chi }_4^0$ we have $p_1\cdot p_2=m_1 E_2$ with

64 $$\begin{aligned} E_2 = \frac{m_1^2 +m_2^2 -M_{h_k}^2}{2m_1}. \end{aligned}$$

Then the decay width of $\tilde{\chi }_4^0\rightarrow \tilde{\chi }_{1}^{0}h_k$ for the production of $h_k=\left\{ h, H \right\}$ equals

65 $$\begin{aligned} \Gamma (\tilde{\chi }_4^0\rightarrow \tilde{\chi }_{1}^{0}h_k)&= \frac{|C_{h_k\tilde{\chi }_4^0\tilde{\chi }_{1}^{0}}|^2}{16 \pi m_{\tilde{\chi }_4^0}^3}((m_{\tilde{\chi }_4^0}+ m_{\tilde{\chi }_1^0})^2 - M_{h_k}^2)\nonumber \\&\quad \times \sqrt{(m_{\tilde{\chi }_4^0}^2-m_{\tilde{\chi }_1^0}^2-M_{h_k}^2)^2 - 4m_{\tilde{\chi }_1^0}^2M_{h_k}^2}\,. \end{aligned}$$

Summing over spins in the final states, the partial decay widths of *h* and *H* into a pair of $\tau$-leptons and the branching ratios are at tree level, improved by 2-loop Higgs masses and total widths from FeynHiggs [48–51],

66 $$\begin{aligned} \Gamma (h_k\rightarrow \tau \tau )&= \frac{1}{\pi }|C_{h_k\tau \tau }|^2 \frac{\left[ \frac{M_{h_k}^2}{4}-m_{\tau }^2 \right] ^{3/2}}{M_{h_k}^2},\nonumber \\ \text {BR}_k&= \frac{\Gamma (h_k\rightarrow \tau ^+\tau ^-)}{\Gamma _{h_k}^\mathrm{{tot}}}, \end{aligned}$$

where $\Gamma _{h_k}^\mathrm{{tot}}$ is the total width. Loop-corrections to the partial decay widths of these subprocesses are calculated with FormCalc [43–47] in Sect. 9.1.

### Unsquared matrix elements

For the calculation of the interference term according to Eq. (24), we need the on-shell matrix elements of the production and decay part. Instead of evaluating absolute values of squared, spin-averaged matrix elements by applying spinor traces, we now aim at expressing the unsquared matrix elements explicitly in order to evaluate them on the appropriate mass shell. Therefore, we need to represent spin wave functions in terms of energy and mass. Following Ref. [71], a Dirac spinor with an arbitrary helicity can be written as

67 $$\begin{aligned} u(p)= \left( \begin{array}{c} \sqrt{E+m}~\chi \\ \sqrt{E-m}~\vec {\sigma }\cdot \hat{p}~\chi \end{array} \right) , \end{aligned}$$

where $\chi$ is a two-component spinor. The eigenstates $\chi$ of the helicity operator $\mathbf {\sigma }\cdot \hat{p}$ with eigenvalues $\lambda =\pm \frac{1}{2}$ satisfy

68 $$\begin{aligned} \left[ \frac{1}{2} \mathbf {\sigma }\cdot \hat{p}\right] \chi _{\lambda } =\lambda \chi _{\lambda }. \end{aligned}$$

For the unit vector $\hat{p}$ in the direction parametrised by the polar angle $\theta$ and azimuthal angle $\phi$ relative to the *z*-axis, the two-component spinors are expressed as

69 $$\begin{aligned} \chi _{+1/2}(\hat{p}) = \left( \begin{matrix} \cos \frac{\theta }{2}\\ e^{i\phi }\sin \frac{\theta }{2} \end{matrix} \right) , \quad \chi _{-1/2}(\hat{p}) = \left( \begin{matrix} -e^{-i\phi }\sin \frac{\theta }{2}\\ \cos \frac{\theta }{2} \end{matrix} \right) . \end{aligned}$$

For the specific choice of $\mathbf {p}\propto e_z$ we have $\theta =0$ and $\phi$ is arbitrary so that it can be set to 0. Thus, the 2-spinors take the simpler form

70 $$\begin{aligned} \chi _{1/2}(\hat{p}=e_z) = e_1\equiv \left( \begin{matrix} 1\\ 0 \end{matrix} \right) ,\quad \chi _{-1/2}(\hat{p}=e_z) = e_2\equiv \left( \begin{matrix} 0\\ 1 \end{matrix} \right) . \end{aligned}$$

We label the unit vectors in space as $\left\{ e_x, e_y, e_z\right\}$ whereas the basis of the 2-spinors is denoted by $\left\{ e_1, e_2\right\}$. The two-component spinors in the opposite momentum direction $\hat{p}=-e_z$ are constructed using

71 $$\begin{aligned} \chi _{-\lambda }(-\hat{p}) = \xi _{\lambda }\chi _{\lambda }(\hat{p}) \end{aligned}$$

from Ref. [71] with $\xi _{\lambda }=1$ in the Jacob-Wick convention for a second particle spinor [72], resulting in

72 $$\begin{aligned} \chi _{+1/2}(-e_z) =e_2, \, \chi _{-1/2}(-e_z) =e_1. \end{aligned}$$

Defining $\epsilon _+ := \sqrt{E+m}$ and $\epsilon _- := \sqrt{E-m}$ for a simpler notation, we can rewrite the particle and antiparticle four-component spinors as

73 $$\begin{aligned}&u_{\lambda }(p) = \begin{pmatrix} \epsilon _+ \chi _{\lambda }(\hat{p})\\ 2\lambda \,\epsilon _- \chi _{\lambda }(\hat{p}) \end{pmatrix}= \begin{pmatrix} \rho ^{\lambda } \\ \psi ^{\lambda }\end{pmatrix}, \nonumber \\&v_{\lambda }(p) = \begin{pmatrix} \epsilon _- \chi _{-\lambda }(\hat{p})\\ -2\lambda \,\epsilon _+ \chi _{-\lambda }(\hat{p}) \end{pmatrix} = \begin{pmatrix} \sigma ^{\lambda } \\ \varphi ^{\lambda } \end{pmatrix}. \end{aligned}$$

Here we introduced the nomenclature $\rho / \psi$ for the upper/lower 2-spinor within a particle 4-spinor *u* and likewise $\sigma / \varphi$ for an antiparticle *v*. For later use, we now list the combinations of $\lambda =\pm \frac{1}{2}$ and $\hat{p}=\pm e_z$ explicitly:

74 $$\begin{aligned}&u_+(e_z)= \begin{pmatrix} \epsilon _{+}e_1\\ \epsilon _{-}e_1 \end{pmatrix},&u_-(e_z)= \begin{pmatrix} \epsilon _{+}e_2\\ -\epsilon _{-}e_2 \end{pmatrix},\nonumber \\&u_+(-e_z)= \begin{pmatrix} \epsilon _{+}e_2\\ \epsilon _{-}e_2 \end{pmatrix},&u_-(-e_z)= \begin{pmatrix} \epsilon _{+}e_1\\ -\epsilon _{-}e_1 \end{pmatrix},\nonumber \\&v_+(e_z)= \begin{pmatrix} \epsilon _{-}e_2\\ -\epsilon _{+}e_2 \end{pmatrix},&v_-(e_z)= \begin{pmatrix} \epsilon _{-}e_1\\ \epsilon _{+}e_1 \end{pmatrix},\nonumber \\&v_+(-e_z)= \begin{pmatrix} \epsilon _{-}e_1\\ -\epsilon _{+}e_1 \end{pmatrix},&v_-(-e_z)= \begin{pmatrix} \epsilon _{-}e_2\\ \epsilon _{+}e_2 \end{pmatrix}. \end{aligned}$$

In the following, we will apply this formalism to Higgs production and decay within our example process.

**Higgs production** As illustrated in Fig. 2b, the incoming spinor $u_1$ (in the example case $\tilde{\chi }_4^0$) decays into $u_2$ ($\tilde{\chi }_{1}^{0}$) and a scalar (*h* / *H*). The matrix element $\mathcal {P}$ of this production process is decomposed into a right- and left-handed part,

75 $$\begin{aligned} \mathcal {P}= \bar{u}_2 C_R\omega _R u_1 + \bar{u}_2 C_L\omega _L u_1 , \end{aligned}$$

where $C_{R/L}$ are form factors. Using $\gamma ^{0}, \gamma ^{5}$ in the Dirac representation, and the 2-spinor notation introduced in Eq. (73), we calculate the spinor chains with arbitrary helicity of $\lambda _1, \lambda _2=\pm \frac{1}{2}$,

76 $$\begin{aligned} p_R&:= \bar{u}_2\omega _R u_1= \frac{1}{2}(\rho _2^{*}-\psi _2^{*})(\rho _1+\psi _1),\end{aligned}$$

77 $$\begin{aligned} p_L&:= \bar{u}_2\omega _L u_1= \frac{1}{2}(\rho _2^{*}+\psi _2^{*})(\rho _1-\psi _1). \end{aligned}$$

Given the 2-body decay in the rest frame of particle 1, it follows that $E_1=m_1$ and consequently $\epsilon _{-}=0,~\psi _1=0$. In order to obtain the helicity matrix elements $p_{R/L}^{\lambda _2\lambda _1}$, we insert the explicit spinors from Eq. (74) into the generic Eq. (77):

78 $$\begin{aligned} p_R^{++}&= \bar{u}_{2+} \omega _R u_{1+} = \frac{1}{2}(\epsilon _{2+}-\epsilon _{2-})\epsilon _{1+}~e_1\cdot e_1\nonumber \\&= \frac{1}{2}\left( \sqrt{E_2+m_2}-\sqrt{E_2-m_2}\right) \sqrt{2m_1},\nonumber \\ p_L^{++}&= \frac{1}{2}\left( \sqrt{E_2+m_2}+\sqrt{E_2-m_2}\right) \sqrt{2m_1},\nonumber \\ p_R^{--}&= p_L^{++},\quad p_L^{--} = p_R^{++},\nonumber \\ p_{R/L}^{+-}&= p_{R/L}^{-+} \propto e_1\cdot e_2 \equiv 0 . \end{aligned}$$

Since the helicity matrix elements are real, their complex conjugates $p_{R/L}^{*}=\bar{u}_1\omega _{L/R}u_2$ are equal to the results in Eq. (78). The products of matrix elements are summed over all helicity combinations (but no averaging is done yet), with $i,j\in \left\{ R,L\right\}$, leading to^3^

79 $$\begin{aligned} A_{ij} := \sum _{\lambda _1,\lambda _2=\pm 1/2} p_i\cdot p_j^{*}, \end{aligned}$$

80 $$\begin{aligned} A_{RR}&= A_{RR}^{++} + A_{RR}^{--} = 2m_1E_2 = m_1^{2}+m_2^{2}-M^{2}, \nonumber \\ A_{LL}&= A_{LL}^{++} + A_{LL}^{--} = A_{RR},\nonumber \\ A_{RL}&= A_{RL}^{++} + A_{RL}^{--} = 2m_1m_2, \nonumber \\ A_{LR}&= A_{LR}^{++} + A_{LR}^{--} = A_{RL} , \end{aligned}$$

where the energy relation of a 2-body decay with $m_1\rightarrow \left\{ m_2, M\right\}$ was applied:

81 $$\begin{aligned} E_2 = \frac{m_1^{2}+m_2^{2}-M^{2}}{2m_1} . \end{aligned}$$

Finally, the squared production matrix element is constructed as

82 $$\begin{aligned} \mathcal {P}\mathcal {P}^{*}&=\sum _{i,j=R,L}C_i C_j^{*} A_{ij} \!=\! (|C_R|^{2}+|C_L|^{2})(m_1^{2}+m_2^{2}-M^{2})\nonumber \\&\quad +(C_R C_L^{*}+C_L C_R^{*})\,2m_1 m_2 . \end{aligned}$$

If the left- and right-handed form factors coincide ($C_L=C_R\equiv C$), Eq. (82) is reduced to

83 $$\begin{aligned} (\mathcal {P}\mathcal {P}^{*})_C&= 2|C|^{2}((m_1+m_2)^{2}-M^{2}). \end{aligned}$$

However, in the interference term we need the product $\mathcal {P}_h \mathcal {P}_H^{*}$ with different Higgs masses in $E_2$ from Eq. (81). This distinction leads to

84 $$\begin{aligned} A_{ij}&= \sum _{\lambda _1,\lambda _2=\pm 1/2}p_i^{h}p_j^{H*},\end{aligned}$$

85 $$\begin{aligned} A_{RR}&= A_{LL} = m_1(\epsilon _{2+}^{h}\,\epsilon _{2+}^{H}+\epsilon _{2-}^{h}\epsilon _{2-}^{H}),\end{aligned}$$

86 $$\begin{aligned} A_{RL}&= A_{LR} = m_1(\epsilon _{2+}^{h}\,\epsilon _{2+}^{H}-\epsilon _{2-}^{h}\epsilon _{2-}^{H}). \end{aligned}$$

As before, we give the resulting product of matrix elements for the independent $C_{R/L}$ and for simpler use in the special case of $C_{R/L}\equiv C$,

87 $$\begin{aligned} \mathcal {P}_h\mathcal {P}_H^{*}&= (C_R^{h}C_R^{H*}+C_L^{h}C_L^{H*})m_1(\epsilon _{2+}^{h}\,\epsilon _{2+}^{H}+\epsilon _{2-}^{h}\epsilon _{2-}^{H}) \nonumber \\&\quad + (C_R^{h} C_L^{H*}+C_L^{h} C_R^{H*})m_1(\epsilon _{2+}^{h}\,\epsilon _{2+}^{H}-\epsilon _{2-}^{h}\epsilon _{2-}^{H})\nonumber \\\end{aligned}$$

88 $$\begin{aligned}&\mathop {\longrightarrow }\limits ^{C} 4C^{h}C^{H*} m_1 \epsilon _{2+}^{h}\epsilon _{2+}^{H} = 2C^{h}C^{H*}\nonumber \\&\quad \times \sqrt{(m_1+m_2)^{2}-M_h^{2}}\,\sqrt{(m_1+m_2)^{2}-M_H^{2}}. \end{aligned}$$

Eq. (87) shows that the method of on-shell matrix elements enables us to distinguish between different masses of the intermediate particles, in this example $M_h$ and $M_H$.

**Table 1:** Parameter settings of the modified $M_h^\mathrm{{max}}$ scenario in the numerical analysis. A value in brackets indicates that the parameter is varied around this central value

$M_1$	$M_2$	$M_3$	$M_\mathrm{{SUSY}}$	$X_t$	$\mu$	$t_{\beta }$	$M_{H^{\pm }}$
100 GeV	200 GeV	800 GeV	1 TeV	2.5 TeV	200 GeV	50	(153 GeV)

**Higgs decay** In the decay of a Higgs boson into a pair of fermions, the representation of antiparticle spinors from Eq. (74) is also needed. Furthermore, the fermions are generated back to back in the rest frame of the decaying Higgs boson. So if we align the momentum direction of the particle spinor $u_4$ with the *z*-axis, $\hat{p}_4=e_z$, the momentum of the antiparticle spinor $v_3$ points into the direction of $\hat{p}_3=-e_z$.

Analogously to Eq. (75), the decay matrix element is in general composed of a left- and right-handed part,

89 $$\begin{aligned} \mathcal {D}&= \bar{u}_4 C_R\omega _R v_3 + \bar{u}_4 C_L\omega _L v_3 ,\end{aligned}$$

90 $$\begin{aligned} d_R&:= \bar{u}_4(e_z) \omega _R v_3(-e_z) = \frac{1}{2}(\rho _4^{*}-\psi _4^{*})(\sigma _3+\varphi _3),\end{aligned}$$

91 $$\begin{aligned} d_L&:= \bar{u}_4(e_z) \omega _R v_3(-e_z) = \frac{1}{2}(\rho _4^{*}+\psi _4^{*})(\sigma _3-\varphi _3). \end{aligned}$$

With the mass *M* of the decaying Higgs boson, the fermion masses $m_3=m_4\equiv m$ and the resulting energies $E_3=E_4\equiv \frac{M}{2}$, the spinor chains $d_R, d_L$ are now calculated for all helicity configurations of $\lambda _3, \lambda _4 =\pm \frac{1}{2}$,

92 $$\begin{aligned} d_R^{++}&= d_L^{--} = \sqrt{E^{2}-m^{2}}-E, \nonumber \\ d_L^{++}&= d_R^{--} = \sqrt{E^{2}-m^{2}}+E, \, d_{R/L}^{+-} = d_{R/L}^{-+} =0. \end{aligned}$$

Summing over all helicity combinations, we obtain

93 $$\begin{aligned} A_{RR} = A_{LL} = M^{2}-2m^{2},\quad A_{RL} = A_{LR} = -2m^{2}. \end{aligned}$$

So the product of on-shell decay matrix elements results in

94 $$\begin{aligned} \mathcal {D}\mathcal {D}^{*}=(|C_R|^{2}+|C_L|^{2})(M^{2}-2m^{2})-(C_RC_L^{*}+C_LC_R^{*})2m^{2} . \end{aligned}$$

In case of identical left- and right-handed couplings *C* of the decay vertex, Eq. (94) simplifies to

95 $$\begin{aligned} \mathcal {D}\mathcal {D}^{*} = 2|C|^{2} (M^{2}-4m^{2}). \end{aligned}$$

As in the production case, we are interested in the contribution to the on-shell interference term, so we distinguish between $E_h= \frac{M_h}{2}$ and $E_H= \frac{M_H}{2}$,

96 $$\begin{aligned} A_{RR}&= A_{LL} =2 \left( \sqrt{(E_h^{2}-m^{2})(E_H^{2}-m^{2})} +E_hE_H\right) ,\nonumber \\ A_{RL}&= A_{LR} =2 \left( \sqrt{(E_h^{2}-m^{2})(E_H^{2}-m^{2})} -E_hE_H\right) . \end{aligned}$$

Finally, the product of decay matrix elements with different masses reads

97 $$\begin{aligned} \mathcal {D}_h\mathcal {D}_H^{*}&= 2(C_R^{h}C_R^{H*}+C_L^{h}C_L^{H*}) \nonumber \\&\qquad \times \,\left( \sqrt{(E_h^{2}-m^{2})(E_H^{2}-m^{2})} +E_hE_H\right) \nonumber \\&\quad + 2(C_R^{h}C_L^{H*}+C_L^{h}C_R^{H*}) \nonumber \\&\qquad \times \,\left( \sqrt{(E_h^{2}-m^{2})(E_H^{2}-m^{2})} -E_hE_H\right) \end{aligned}$$

98 $$\begin{aligned}&\quad \mathop {\longrightarrow }\limits ^{C}8C^{h}C^{H*}\sqrt{\left( \frac{M_h^{2}}{4}-m^{2}\right) \left( \frac{M_H^{2}}{4}-m^{2}\right) }, \end{aligned}$$

where the last line applies for identical *L* / *R* form factors.

The outcome of the explicit spinor representations in the context of factorising a longer process into production and decay is the possibility to express the interference term with on-shell matrix elements depending on the mass of the intermediate particle. The method was here introduced in a generic way and then applied to the example of Higgs production and decay with two external fermions in each subprocess in the rest frames of the decaying particles.

## Numerical evaluation at lowest order

### Modified $M_h^\mathrm{{max}}$ scenario

In order to apply the gNWA to the example process of $\tilde{\chi }_4^0\rightarrow \tilde{\chi }_{1}^{0}\,h/H\rightarrow \tilde{\chi }_{1}^{0}\,\tau ^{+}\tau ^{-}$ numerically, we specify a scenario. In this study, we restrict the MSSM parameters to be real so that there is no new source of $\mathcal {CP}$-violation compared to the SM and only the two $\mathcal {CP}$-even neutral Higgs bosons, *h* and *H*, mix and interfere with each other. The aim here is not to determine the parameters which are currently preferred by recent limits from experiments, but to provide a setting in which interference effects between *h* and *H* become large in order to investigate the performance of the generalised narrow-width approximation for this simple example process.

The $M_h^\mathrm{{max}}$ scenario [73, 74] is defined such that the loop corrections to the mass $M_h$ reach their maximum for fixed $\tan \beta ,~M_A$ and $M_\mathrm{{SUSY}}$. This requires a large stop mixing, i.e. a large off-diagonal element $X_t$ of the stop mixing matrix in Eq. (55). A small mass difference $\Delta M \equiv M_H-M_h$ requires a rather low value of $M_A$, or equivalently $M_{H^{\pm }}$, and a high value of $\tan \beta$. On the other hand, $\tan \beta$ must not be chosen too large because otherwise the bottom Yukawa coupling would be enhanced to an non-perturbative value. We modify the $M_h^\mathrm{{max}}$ scenario such that $M_h$ is not maximised, but the mass difference $\Delta M$ is reduced by raising $X_t$. As one of the Higgs sector input parameters, we choose $M_H^{\pm }$ for a later extension to $\mathcal {CP}$-violating mixings instead of $M_A$, which is more commonly used in the MSSM with real parameters. The charged Higgs mass is scanned over the range $M_{H^{\pm }}\in$[151 GeV, 155 GeV]. The other parameters are defined in Table 1, and we assume universal trilinear couplings $A_f=A_t$.

**Fig. 3:**
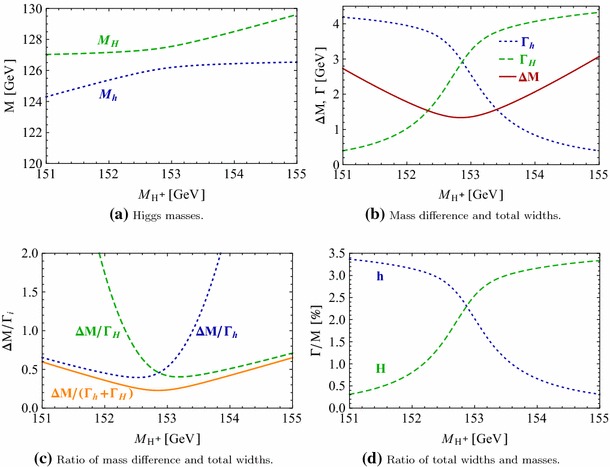
Higgs masses and widths from FeynHiggs [48–51] including dominant 2-loop corrections in the modified $M_{h}^\mathrm{{max}}$ scenario. **a** Higgs masses $M_h$ (*blue*, *dotted*) and $M_H$ (*green*, *dashed*). **b** Mass difference $\Delta M\equiv M_H-M_h$ (*red*) compared to total widths $\Gamma _h$ (*blue*, *dotted*) and $\Gamma _H$ (*green*, *dashed*). **c** Mass difference $\Delta M$ divided by total width of *h* (*blue*, *dotted*), *H* (*green*, *dashed*) and sum of both widths (*orange*). **d** Ratio $\Gamma _i/M_i$ for *h* (*blue*, *dotted*) and *H* (*green*, *dashed*)

Under variation of the input Higgs mass $M_{H^{\pm }}$, the resulting masses and widths of the interfering neutral Higgs bosons *h*, *H* change as shown in Fig. 3 with results from FeynHiggs [48–51] including dominant 2-loop corrections. Figure 3(a) displays the dependence of the masses of *h* (blue, dotted) and *H* (green, dashed) on $M_{H^{\pm }}$. Within the analysed parameter range of $M_{H^{\pm }}= 151 \cdots 155\,$GeV, their mass difference $\Delta M$ (red) in Fig. 3b is around its minimum at $M_{H^{\pm }}\simeq 153\,$GeV smaller than both total widths $\Gamma _h$ (blue, dotted) and $\Gamma _H$ (green, dashed). While $\Gamma _h$ decreases, $\Gamma _H$ increases with increasing $M_{H^{\pm }}$. This is caused by a change of the predominantly diagonal or off-diagonal structure of the $\hat{\mathbf{Z }}$-matrix which has a cross-over around $M_{H^{\pm }}\simeq 153$ GeV in this scenario. Since both widths contribute to the overlap of the two resonances, the ratio $R_{M\Gamma }=\Delta M/(\Gamma _h+\Gamma _H)$ gives a good indication of the parameter region of most significant interference. This is visualised (in orange) in Fig. 3c and compared to the ratios $\Delta M/\Gamma _h$ (blue, dotted) and $\Delta M/\Gamma _H$ (green, dashed), which only take one of the widths into account and are therefore a less suitable criterion for the importance of the interference term. Figure 3d presents the ratio $\Gamma _i/M_i$ for $i=h$ (blue, dotted) and *H* (green, dashed) as a criterion for a *narrow* width. Both ratios lie in the range of about 0.5–$3.5\,\%$, and this represents the expected order of the NWA uncertainty.

### Results for tree level process $\tilde{\chi }_4^0\rightarrow \tilde{\chi }_{1}^{0}\,h/H\rightarrow \tilde{\chi }_{1}^{0}\,\tau ^{+}\tau ^{-}$

In order to understand the possible impact of interference terms, we confront the prediction of the standard NWA with the 3-body decay width of our example process $\tilde{\chi }_4^0\rightarrow \tilde{\chi }_{1}^{0}\tau ^{+}\tau ^{-}$ at the tree level (improved by 2-loop predictions for the masses, widths and $\hat{\mathbf{Z }}$-factors) in the modified $M_h^\mathrm{{max}}$ scenario.

**Fig. 4:**
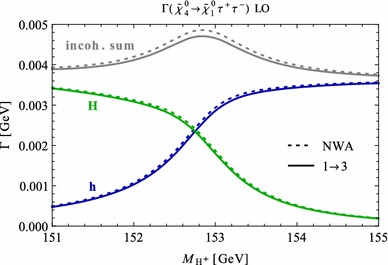
The 1$\rightarrow$3 decay width (*solid*) of $\tilde{\chi }_4^0\rightarrow \tilde{\chi }_{1}^{0}\tau ^{+}\tau ^{-}$ at tree level with separate contributions from *h* (*blue*), *H* (*green*) and their incoherent sum (*grey*) confronted with the sNWA (*dotted*)

First of all, we verify that the other conditions from Sect. 2.2 for the NWA are met. The widths of the involved Higgs bosons do not exceed $3.5\,\%$ of their masses, hence they can be considered *narrow* (see Fig. 3). At tree level, there are no unfactorisable contributions so that the scalar propagator is separable from the matrix elements. Besides, our scenario is far away from the production and decay thresholds since $M_{h_k}\gg 2m_{\tau }$ holds independently of the parameters, and with neutralino masses of $m_{\tilde{\chi }_4^0} \simeq 264.9\,$GeV and $m_{\tilde{\chi }_{1}^{0}}\simeq 92.6\,$GeV, also $m_{\tilde{\chi }_4^0} - (m_{\tilde{\chi }_{1}^{0}}+M_{h_k})> 32\,$GeV does not violate the threshold condition. The neutralino masses are independent of $M_{H^{\pm }}$. Thus, the NWA is applicable for the individual contributions of *h* and *H*, so the factorised versions

99 $$\begin{aligned} \Gamma ^{i}_\mathrm{{NWA}}:= \Gamma _{P_i}(\tilde{\chi }_4^0\rightarrow \tilde{\chi }_{1}^{0}h_i)\,\text {BR}_i(h_i\rightarrow \tau ^{+}\tau ^{-}) \end{aligned}$$

should agree with the separate terms of the 3-body decays via the exchange of only one of the Higgs bosons, $h_i$,

100 $$\begin{aligned} \Gamma ^{i}_{1\rightarrow 3}:=\Gamma (\tilde{\chi }_4^0\mathop {\rightarrow }\limits ^{h_i}\tilde{\chi }_{1}^{0}\tau ^{+}\tau ^{-}) \end{aligned}$$

within the uncertainty of $\mathcal {O}\left( \frac{\Gamma _{h_i}}{M_{h_i}}\right)$. This is tested in Fig. 4. The blue lines compare $\Gamma ^{h}_{1\rightarrow 3}$ (solid) with the factorised process $\Gamma ^{h}_\mathrm{{NWA}}$ (dotted), the green lines represent the corresponding expressions for *H*. The standard narrow-width approximation is composed of the *incoherent* sum of both factorised processes, i.e.,

101 $$\begin{aligned} \Gamma _\mathrm{{sNWA}}= \Gamma _{P_h}\,\text {BR}_h + \Gamma _{P_H}\,\text {BR}_H. \end{aligned}$$

This is confronted with the incoherent sum of the 3-body decays which are only *h*-mediated or *H*-mediated. For a direct comparison with the sNWA, the interference term is not included,

102 $$\begin{aligned} \Gamma ^\mathrm{{incoh}}_{1\rightarrow 3} = \Gamma ^{h}_{1\rightarrow 3}+\Gamma ^{H}_{1\rightarrow 3}. \end{aligned}$$

The sNWA (dotted) and the incoherent sum of the 3-body decay widths are both shown in grey. Their relative deviation of 0.8–3.3 % is of the order of the ratio $\Gamma /M$ from Fig. 3d. Consequently, the NWA is applicable to the terms of the separate *h* / *H*-exchange within the expected uncertainty.

However, the fifth condition in Sect. 2.2 concerns the absence of a large interference with other diagrams. But with $\Delta M<\Gamma _h+\Gamma _H$ throughout the analysed parameter range (see Fig. 3c), we expect a sizeable interference effect in this scenario owing to a considerable overlap of the Breit–Wigner propagators and a sizeable mixing between *h* and *H*. Since the masses and widths of the interfering Higgs bosons depend on $M_{H^{\pm }}$, the size of the interference term varies with the input charged Higgs mass. Based on the minimum of the ratio $R_{\Gamma M}=\Delta M/(\Gamma _h+\Gamma _H)$ and a significant mixing between *h* and *H*, we expect the most significant interference contribution near $M_{H^{\pm }}=153$ GeV.

**Fig. 5:**
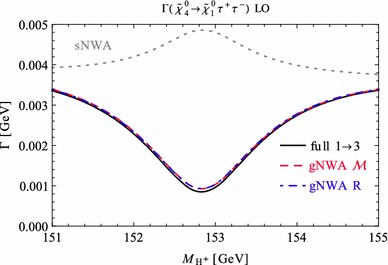
The 1$\rightarrow$3 decay width of $\tilde{\chi }_4^0\rightarrow \tilde{\chi }_{1}^{0}\tau ^{+}\tau ^{-}$ at tree level with contributions from *h*, *H* including their interference (*black*) confronted with the NWA: sNWA without the interference term (*grey*, *dotted*), gNWA including the interference term based on on-shell matrix elements denoted by $\mathcal {M}$ (*red*, *dashed*) and on the *R*-factor approximation denoted by *R* (*blue*, *dash-dotted*)

Figure 5 presents the partial decay width $\Gamma (\tilde{\chi }_4^0\rightarrow \tilde{\chi }_{1}^{0}\tau ^{+}\tau ^{-})$ in dependence of the input Higgs mass $M_{H^{\pm }}$. In the sNWA (grey), the interference term is absent. In contrast, the full 3-body decay^4^ (black) takes the *h* and *H* propagators and their interference into account.

Comparing the prediction of the sNWA with the full 3-body decay width reveals an enormous discrepancy between both results, especially in the region of the smallest ratio $R_{\Gamma M}$ around $M_{H^{\pm }}\simeq 153\,$GeV, due to a large negative interference term. Consequently, the NWA in its standard version is insufficient in this parameter scenario.

In the generalised narrow-width approximation, on the other hand, the sNWA is extended by incorporating the on-shell interference term. The red line indicates the prediction of the complete process in the gNWA using the on-shell evaluation of unsquared matrix elements in the interference term as derived conceptually in Eq. (24) and explicitly in Sect. 5.3. Furthermore, the blue line demonstrates the result of the gNWA using the additional approximation of interference weight factors *R* defined in Eq. (38). While the sNWA overestimates the full result by a factor of up to 5.5 on account of the neglected destructive interference, both variants of the gNWA result in a good approximation of the full 3-body decay width.

The slight relative deviation between either form of the gNWA and the full result amounts to $(\Gamma _\mathrm{{gNWA}}-\Gamma _{1\rightarrow 3})/\Gamma _\mathrm{{sNWA}}\simeq 0.4{-}1.7\,\%$ if normalised to the sNWA and to $(\Gamma _\mathrm{{gNWA}}-\Gamma _{1\rightarrow 3})/\Gamma _{1\rightarrow 3}\simeq 0.5{-}9.2\,\%$ if normalised to the 3-body decay width. The largest relative deviation between $\Gamma _\mathrm{{gNWA}}$ and $\Gamma _{1\rightarrow 3}$ arises in the region where the reference value $\Gamma _{1\rightarrow 3}$ itself is very small so that a small deviation has a pronounced relative effect. This uncertainty, however, is not intrinsically introduced by the approximated interference term, but it stems from the factorised constituents $\Gamma _\mathrm{{NWA}}^{h},~\Gamma _\mathrm{{NWA}}^{H}$ already present in the sNWA, see Fig. 4.

## Application of the gNWA to the loop level

Motivated by the good performance of the gNWA at the tree level, in this section we investigate the application of the generalised narrow-width approximation at the loop level by incorporating 1-loop corrections of the production and decay part into the predictions. Before treating the full 3-body decay width at the next-to leading order (NLO) in Sect. 8, we will start with the method of on-shell matrix elements in Sect. 7.1 and turn to the R-factor approximation in Sect. 7.2.

At the 1-loop level we write the product of the production cross-section times partial decay width in the standard NWA as

103 $$\begin{aligned} \sigma _{P} \cdot \text {BR} \longmapsto \frac{\sigma _{P}^{1}\Gamma _{D}^{0} + \sigma _{P}^{0}\Gamma _{D}^{1}}{\Gamma ^\mathrm{{tot}}}, \end{aligned}$$

where the total width is obtained from FeynHiggs [48–51] incorporating corrections up to the 2-loop level as in the definition of the branching ratio and in the Breit–Wigner propagator. While restricting the numerator of Eq. (103) formally to one-loop order to enable a consistent comparison with the full process, at the end (in Sect. 9.4) all constituents of the NWA will be used at the highest available precision, i.e. $\sigma _{P}^\mathrm{{best}} \cdot \text {BR}^\mathrm{{best}}$ for the most advanced prediction with the branching ratio obtained from FeynHiggs.

### On-shell matrix elements at 1-loop order

In analogy to the procedure in Sect. 3.2 at the tree level, on-shell matrix elements are used here in the 1-loop expansion. Special attention must be paid to the cancellation of infrared (IR) divergences from virtual photons (or gluons) in 1-loop matrix elements and real photon (gluon) emission off charged external legs. In preparation for the example process $\tilde{\chi }_4^0\rightarrow \tilde{\chi }_{1}^{0}\,h/H\rightarrow \tilde{\chi }_{1}^{0}\,\tau ^{+}\tau ^{-}$ (see Sect. 5), we focus on IR-divergences from photons in loops of the decay part and soft final state photon radiation.

The aim is to approximate only the 1-loop contribution, but to keep the full momentum dependent expression at the Born level with $\mathcal {M}_i^{0} = \mathcal {M}_i^{0}(q^{2})$,

104 $$\begin{aligned} |\mathcal {M}^{0}|^{2}= |\mathcal {M}_h^{0}|^{2} + |\mathcal {M}_H^{0}|^{2} + 2\mathrm{Re}[\mathcal {M}_h^{0}\mathcal {M}_H^{0*}]. \end{aligned}$$

In contrast, the 1-loop matrix elements are factorised into the on-shell production and decay parts times the momentum dependent Breit–Wigner propagator $\Delta _i^{\text {BW}}\equiv \Delta _i^{\text {BW}}(q^{2})$. The squared matrix elements are expanded up to the 1-loop order. Since the emission of soft real photons is proportional to the Born contribution, the virtual contribution is supplemented by the absolute value squared of the tree-level matrix element, multiplied by the QED-factor $\delta _{\text {SB}}$ of soft bremsstrahlung [75, 76],

105 $$\begin{aligned}&2\mathrm{Re}[\mathcal {M}^{0}\mathcal {M}^{1*}] + \delta _{\mathrm{SB}} |\mathcal {M}^{0}|^{2} \nonumber \\&\simeq 2\text {Re}[(\mathcal {P}_h^{1}\mathcal {D}_h^{0}+ \mathcal {P}_h^{0}\mathcal {D}_h^{1} + \delta _{\text {SB}} \mathcal {P}_h^{0}\mathcal {D}_h^{0})\mathcal {P}_h^{0*}\mathcal {D}_h^{0*} \cdot |\Delta _h^{BW}|^{2}]\nonumber \\&\quad +2\text {Re}[(\mathcal {P}_H^{1}\mathcal {D}_H^{0}\!+\! \mathcal {P}_H^{0}\mathcal {D}_H^{1} \!+\! \delta _{\text {SB}} \mathcal {P}_H^{0}\mathcal {D}_H^{0})\mathcal {P}_H^{0*}\mathcal {D}_H^{0*}\cdot |\Delta _H^{\text {BW}}|^{2}]\nonumber \\&\quad +2\text {Re}[\lbrace (\mathcal {P}_h^{1}\mathcal {D}_h^{0}+\mathcal {P}_h^{0}\mathcal {D}_h^{1})\mathcal {P}_H^{0*}\mathcal {D}_H^{0*}+\mathcal {P}_h^{0}\mathcal {D}_h^{0}(\mathcal {P}_H^{1*}\mathcal {D}_H^{0*}\nonumber \\&\quad + \mathcal {P}_H^{0*}\mathcal {D}_H^{1*}) + \delta _{\text {SB}}\, \mathcal {P}_h^{0}\mathcal {D}_h^{0}\mathcal {P}_H^{0*}\mathcal {D}_H^{0*} \rbrace \cdot \Delta _h^{\text {BW}}\Delta _H^{\text {BW}*}]. \end{aligned}$$

The first line of Eq. (105) represents the pure contribution from *h*, factorised into production and decay, the second line accordingly for *H*. The third and fourth lines constitute the 1-loop and bremsstrahlung interference term as the product of *h*- and *H*-matrix elements and Breit–Wigner propagators. For a consistent comparison with the full 1-loop result, each term is restricted to 1-loop corrections in only one of the matrix elements.

The 1-loop prediction of the full process in the approximation of on-shell matrix elements consists – besides the Born cross section without an approximation^5^ – of the squared contribution of *h* and *H* and the interference term $\sigma _{\mathcal {M}}^\mathrm{{int}1}$ at the strict 1-loop level,^6^

106 $$\begin{aligned} \sigma _{\mathcal {M}}^{1}&= \sigma _\mathrm{{full}}^{0} + \frac{\sigma _{P_h}^{1}\Gamma _{D_h}^{0}+\sigma _{P_h}^{0}\Gamma _{D_h}^{1}}{\Gamma _h^\mathrm{{tot}}}\nonumber \\&\quad + \frac{\sigma _{P_H}^{1}\Gamma _{D_H}^{0}+\sigma _{P_H}^{0}\Gamma _{D_H}^{1}}{\Gamma _H^\mathrm{{tot}}} +\sigma _{\mathcal {M}}^\mathrm{{int}1}, \end{aligned}$$

107 $$\begin{aligned} \sigma _{\mathcal {M}}^\mathrm{{int}1}&=\frac{2}{F}\text {Re}\left\{ \int \frac{\mathrm{d}q^{2}}{2\pi }\Delta ^{\text {BW}}_h(q^{2}) \Delta ^{*\text {BW}}_H(q^{2}) \right. \nonumber \\&\quad \times \left( \left[ \int \mathrm{d}\Phi _{P}(q^{2})(\mathcal {P}_{h}^{1}\mathcal {P}_{H}^{0*}+\mathcal {P}_{h}^{0}\mathcal {P}^{1*}_{H})\right] \right. \nonumber \\&\quad \times \left. \left. \left[ \int \mathrm{d}\Phi _{D}(q^{2}) \mathcal {D}_{h}^{0}\mathcal {D}^{0*}_{H}\right] +\left[ \int \mathrm{d}\Phi _{P}(q^{2})\mathcal {P}_{h}^{0}\mathcal {P}^{0*}_{H}\right] \right. \right. \nonumber \\&\quad \times \left. \left. \left[ \int \mathrm{d}\Phi _{D}(q^{2}) (\mathcal {D}_{h}^{1}\mathcal {D}^{0*}_{H}+\mathcal {D}_{h}^{0}\mathcal {D}^{1*}_{H}+\delta _{\text {SB}}\mathcal {D}_{h}^{0}\mathcal {D}^{0*}_{H})\right] \right) \right\} . \end{aligned}$$

For the prediction with the most precise constituents, we use 2-loop branching ratios, $\mathrm {BR}_i^\mathrm{{best}}$. We include also the products of 1-loop matrix elements. Their contribution to the interference term is denoted by $\sigma _{\mathcal {M}}^\mathrm{{int}+}$,

108 $$\begin{aligned} \sigma _{\mathcal {M}}^\mathrm{{int}+}&= \frac{2}{F}\text {Re}\left\{ \int \frac{\mathrm{d}q^{2}}{2\pi }\Delta ^{\text {BW}}_h(q^{2}) \Delta ^{*\text {BW}}_H(q^{2})\right. \nonumber \\&\quad \times \left. \left[ \int \mathrm{d}\Phi _{P}(q^{2}) (\mathcal {P}_h^{1}\mathcal {P}_H^{0*}+\mathcal {P}_h^{0}\mathcal {P}_H^{1*})\right] \right. \nonumber \\&\quad \times \left. \left[ \int \mathrm{d}\Phi _{D}(q^{2}) (\mathcal {D}_h^{1}\mathcal {D}_H^{0*}\!+\!\mathcal {D}_h^{0}\mathcal {D}_H^{1*}\!+\!\delta _{\text {SB}}\mathcal {D}_h^{0}\mathcal {D}_H^{0*})\right] \right\} . \end{aligned}$$

The approximation of the whole process based on on-shell matrix elements and incorporating higher-order corrections wherever possible is denoted by $\sigma _{\mathcal {M}}^{\text {best}}$, which reads then

109 $$\begin{aligned} \sigma _{\mathcal {M}}^{\text {best}}&= \sigma _\mathrm{{full}}^{0}+\sum _{i=h,H}(\sigma _{P_i}^{\text {best}}\text {BR}_i^{\text {best}}-\sigma _{P_i}^{0}\text {BR}_i^{0}) \nonumber \\&\quad + \sigma _{\mathcal {M}}^\mathrm{{int}1}+\sigma _{\mathcal {M}}^\mathrm{{int}+}. \end{aligned}$$

The *best* production cross section $\sigma _{P_i}^{\text {best}}$ and branching ratios $\text {BR}_i^{\text {best}}$ mean the sum of the tree level, strict 1-loop and all available higher-order contribution to the respective quantity. Therefore, the products of tree level production cross sections and branching ratios are subtracted because their unfactorised counterparts are already contained in the full tree level term $\sigma _\mathrm{{full}}^{0}$. If a more precise result of the production cross sections is available, it can be used instead of the explicit 1-loop calculation that was performed in our example process.

#### IR-finiteness of the factorised matrix elements

**On-shell evaluation** The UV-divergences of the virtual corrections are cancelled by the same counterterms as in the full process at 1-loop order. Although it would be technically possible in most processes to compute the full bremsstrahlung term without the NWA, i.e. $\delta _{\text {SB}}\,|\mathcal {M}_\mathrm{{full}}^{0}|^{2}$, the IR-divergences from the on-shell decays need to be exactly cancelled by those from the real photon emission. But the IR-singularities in the sum of the factorised (on-shell) virtual corrections and the momentum-dependent real ones would not match each other. Consequently, the tree level matrix elements are also factorised, and the IR-divergent parts of the 1-loop decay matrix elements $\mathcal {D}_h^{1}(M_h^{2}, \overline{M}^{2}), \mathcal {D}_H^{1}(M_H^{2}, \overline{M}^{2})$ and the soft QED-factor $\delta _{\text {SB}}(\overline{M}^{2})$ have to be calculated at the same mass $\overline{M}=M_h$ or $M_H$. The LO matrix elements are evaluated at their mass-shell, i.e. $\mathcal {D}_i^{0}(M_{h_i}^{2})$. The NLO matrix elements are split into the part containing loop integrals on the one hand and the helicity matrix elements on the other hand. While the individual Higgs masses can be inserted into the finite helicity matrix elements (see Sect. 5.3 ), the loop integrals have to be evaluated at the same mass $\overline{M}^{2}$ as in $\delta _{\text {SB}}$ to preserve the IR-cancellations. Hence, a choice must be made whether to define $\overline{M}=M_h$ or $M_H$. We evaluate the numerical difference in Sect. 9.3.

The production matrix elements are completely evaluated on their respective mass-shells, $\mathcal {P}^{0}_i(M_{h_i}^{2})$ and $\mathcal {P}^{1}_i(M_{h_i}^{2})$. This is possible because the initial state in this example contains only neutral particles. But the calculation can be directly generalised to charged initial states according to the procedure described for the decay matrix elements. The IR-singularities in the product of initial and final state radiation are then cancelled by those from a virtual photon connecting charged legs of the initial and final state. Such non-factorisable contributions can be treated in a pole approximation in analogy to the double-pole approximation (DPA) that has been used for instance for the process $e^{+}e^{-}\rightarrow W^{+}W^{-}\rightarrow 4$ leptons, see Ref. [77]. An alternative approach for the treatment of IR-singularities is formulated in Refs. [4, 37]. There, the singular parts from the real photon contribution are extracted, and the DPA is only applied for those terms which exactly match the singularities from the virtual photons. In our calculation, we do not split up the real corrections in this way, but employ instead the procedure described above. We discuss a possibility of splitting the diagrams with virtual photons into an IR-singular and a finite subgroup in Sect. 7.1.2.

**Cancellation of IR-divergences** According to the Kinoshita–Lee–Nauenberg (KLN) theorem [78, 79], the IR-divergence from a virtual photon is cancelled by the emission of a real photon off a charged particle from the initial or final state, i.e., in our example process as soft bremsstrahlung in the final state of a Higgs decay. We will derive the IR-finiteness of the on-shell matrix elements in analogy to the cancellation of the IR divergencies for the full 3-body decay. Writing the momentum-dependent 3-body matrix elements with the resonant particle either $h_i=h$ or *H* as the sum of the tree level ($\mathcal {M}_{h_i}^{0}$) and virtual ($\mathcal {M}_{h_i}^{v}$) contributions,

110 $$\begin{aligned} \mathcal {M}_{h_i}(q^{2}) = \mathcal {M}_{h_i}^{0}(q^{2})+\mathcal {M}_{h_i}^{v}(q^{2}) , \end{aligned}$$

and adding to the squared matrix element the corresponding contribution from real soft photon ($\mathcal {M}_{h_i}^\mathrm{{Br}}$) radiation, we find

111 $$\begin{aligned}&|\mathcal {M}_{h}+\mathcal {M}_H|^{2}+|\mathcal {M}_{h}^\mathrm{{Br}}+\mathcal {M}_H^\mathrm{{Br}}|^{2} \nonumber \\&\quad = \sum _{h_i=h,H}(|\mathcal {M}_{h_i}|^{2}+|\mathcal {M}_{h_i}^\mathrm{{Br}}|^{2})\nonumber \\&\qquad + 2\text {Re}[\mathcal {M}_{h}\mathcal {M}_{H}^{*}+\mathcal {M}_{h}^\mathrm{{Br}}\mathcal {M}_H^\mathrm{{Br}*}]. \end{aligned}$$

Because the complete sum in Eq. (111) and the individual *h*- and *H*-terms are IR-finite, the interference term must be IR-finite by itself. With the proportionality of the bremsstrahlung contribution to the tree level term,

112 $$\begin{aligned} \mathcal {M}_{h}^\mathrm{{Br}}(q^{2})\mathcal {M}_H^\mathrm{{Br}*}(q^{2}) = \delta _{\text {SB}}(q^{2})\mathcal {M}_h^{0}(q^{2})\mathcal {M}_H^{0*}(q^{2}), \end{aligned}$$

and keeping only the terms of $\mathcal {O}(\alpha )$ relative to the lowest order, the interference term $\text {Int}^{\alpha }(q^{2})$ results in

113 $$\begin{aligned} \text {Int}^{\alpha }(q^{2})&= 2\text {Re}[\mathcal {M}_h(q^{2})\mathcal {M}_H^{*}(q^{2})|_{\alpha }\!+\!\mathcal {M}_{h}^{Br}(q^{2})\mathcal {M}_H^{Br*}(q^{2})]\end{aligned}$$

114 $$\begin{aligned}&= 2\text {Re}[\mathcal {M}_h^{v}(q^{2})\mathcal {M}_H^{0*}(q^{2})+\mathcal {M}_h^{0}(q^{2})\mathcal {M}_H^{v*}(q^{2})\nonumber \\&\quad +\delta _{\text {SB}}(q^{2})\mathcal {M}_h^{0}(q^{2})\mathcal {M}_H^{0*}(q^{2})]. \end{aligned}$$

As described above, the on-shell evaluation is performed at the individual mass $M_{h_i}$ in all production and tree level matrix elements and the helicity elements, whereas the soft photon factor $\delta _{\text {SB}}$ and the 1-loop form factors of the decay are evaluated at the same mass $\overline{M}$ in the on-shell interference term $\text {Int}_{\text {os}}^{\alpha }$ of $\mathcal {O}(\alpha )$ relative to the lowest order,

115 $$\begin{aligned} \text {Int}_{\text {os}}^{\alpha }&= 2\text {Re}[\mathcal {M}_h^{v}(M_h^{2},\overline{M}^{2})\mathcal {M}_H^{0*}(M_H^{2})\nonumber \\&\quad +\mathcal {M}_h^{0}(M_h^{2})\mathcal {M}_H^{v*}(M_H^{2},\overline{M}^{2})\nonumber \\&\quad +\delta _{\text {SB}}(\overline{M}^{2})\mathcal {M}_h^{0}(M_h^{2})\mathcal {M}_H^{0*}(M_H^{2})]\end{aligned}$$

116 $$\begin{aligned}&= 2\text {Re}[\lbrace (\mathcal {P}_h^{v}(M_h^{2})\mathcal {D}_h^{0}(M_h^{2})+\mathcal {P}_h^{0}(M_h^{2})\mathcal {D}_h^{v}(M_h^{2},\overline{M}^{2}))\nonumber \\&\quad \cdot \mathcal {P}_H^{0*}(M_H^{2})\mathcal {D}_H^{0*}(M_H^{2})+\mathcal {P}_h^{0}(M_h^{2})\mathcal {D}_h^{0}(M_h^{2})\nonumber \\&\quad \cdot (\mathcal {P}_H^{v*}(M_H^{2})\mathcal {D}_H^{0*}(M_H^{2})+\mathcal {P}_H^{0*}(M_H^{2})\mathcal {D}_H^{v*}(M_H^{2},\overline{M}^{2})) \nonumber \\&\quad +\delta _{\text {SB}}(\overline{M}^{2})\,\mathcal {P}_h^{0}(M_h^{2})\mathcal {D}_h^{0}(M_h^{2})\,\mathcal {P}_H^{0*}(M_H^{2})\mathcal {D}_H^{0*}(M_H^{2}) \rbrace \nonumber \\&\quad \times \Delta _h(q^{2})\Delta _H^{*}(q^{2})]. \end{aligned}$$

Since the virtual production matrix elements are IR-finite in our example process, we can drop the first term in each of the brackets in the first and second line of Eq. (116) for the discussion of IR-singularities, which are contained in $\text {Int}_{\text {os}}^{\alpha }|_{\text {IR}}$,

117 $$\begin{aligned} \text {Int}_{\text {os}}^{\alpha }|_{\text {IR}}&= 2\mathrm {Re}[\mathcal {P}_h^{0}(M_h^{2})\mathcal {P}_H^{0*}(M_H^{2})\cdot \Delta _h(q^{2})\Delta _H^{*}(q^{2})\nonumber \\&\quad \cdot (\mathcal {D}_h^{v}(M_h^{2},\overline{M}^{2})\mathcal {D}_H^{0*}(M_H^{2})+\mathcal {D}_h^{0}(M_h^{2})\mathcal {D}_H^{v*}(M_H^{2},\overline{M}^{2})\nonumber \\&\quad +\delta _{\text {SB}}(\overline{M}^{2})\mathcal {D}_h^{0}(M_h^{2})\mathcal {D}_H^{0*}(M_H^{2}))]. \end{aligned}$$

Moreover, the $M_{h_i}^{2}$-dependent helicity matrix elements $d_{h_i}(M_{h_i}^{2})$ from Sect. (5.3) can be factored out by $\mathcal {D}_{h_i}=C_{h_i}d_{h_i}$ so that the IR-singularities from $\left. \text {Int}_{\text {os}}^{\alpha }\right| _{\text {IR}}$ can be further extracted:

118 $$\begin{aligned} \text {Int}_{\text {os}}^{\alpha }|_{\text {IR}}&= 2\text {Re}[\mathcal {P}_h^{0}(M_h^{2})\mathcal {P}_H^{0*}(M_H^{2})\nonumber \\&\quad \cdot \Delta _h(q^{2})\Delta _H^{*}(q^{2})\cdot d_h(M_h^{2})\,d_H^{*}(M_H^{2})(C_h^{v}(\overline{M}^{2})C_H^{0*}\nonumber \\&\quad +C_h^{0}C_H^{v*}(\overline{M}^{2})+\delta _{\text {SB}}(\overline{M}^{2})C_h^{0}C_H^{0*})]. \end{aligned}$$

Compared to Eq. (114) which can also be factorised into $q^{2}$-dependent form factors and helicity matrix elements, the structure of the IR-singularities is the same. In Eq. (118), all of those contributions are just evaluated at $\overline{M}^{2}$ instead of $q^{2}$. Hence the cancellation works analogously so that Eq. (115) is an IR-finite formulation of the factorised interference term. Because the $\hat{\mathbf{Z }}$-factors can be factored out in the same way for the on-shell approximation as for the full matrix elements, their inclusion preserves the cancellations of IR-divergences.

#### Separate calculation of photon diagrams

As an alternative to the method described above, it is possible to reduce the number of diagrams whose loop integrals need to be evaluated at the common mass $\overline{M}$ instead of their on-shell mass $M_i$ by splitting the 1-loop decay matrix elements into an IR-finite and an IR-divergent part,

119 $$\begin{aligned} \mathcal {D}_i^{1} = \mathcal {D}_i^{1,\mathrm {no}\gamma } + \mathcal {D}_i^{1,\gamma }. \end{aligned}$$

Both subgroups of diagrams are rendered UV-finite by the corresponding counterterms. Since the diagrams without any photon are already IR-finite, their loop integrals can safely be calculated on-shell, $\mathcal {D}_i^{1,\mathrm {no}\gamma }(M_{h_i}^{2})$. Hence, only the loop-integrals of the photon contribution need to be evaluated at a fixed mass $\overline{M}$, resulting in $\mathcal {D}_i^{1,\gamma }(M_{h_i}^{2}, \overline{M}^{2})$ and $\delta _{\text {SB}}(\overline{M}^{2})$.

If the fixed Higgs mass were inserted into both the loop integrals and the helicity matrix elements, the IR-cancellation would work in the same way as for the unfactorised process, just with the special choice of $q^{2}=\overline{M}^{2}$. In our approach, the helicity matrix elements are determined at the specific masses $M_{h_i}$ as it is demonstrated in Eqs. (87) and (97). Furthermore, those mass values are equal in the matrix elements at lowest and higher orders as loop-corrected masses are used also at the improved Born level. Because the $M_{h_i}$-dependent helicity matrix elements can be factored out, the IR-singularities cancel in the decay contribution to the interference term of $\mathcal {O}(\alpha )$ relative to the lowest order, with $\mathcal {D}_i^{0}$ at $M_{h_i}^{2}$,

120 $$\begin{aligned} \left( \mathcal {D}_h\mathcal {D}_H^{*}\right) ^{\alpha }&=\mathcal {D}_h^{1,\gamma }(M_h^{2},\overline{M}^{2})\mathcal {D}_H^{0*}+\mathcal {D}_h^{0}\mathcal {D}_H^{1,\gamma *}(M_H^{2},\overline{M}^{2}) \nonumber \\&\quad + \delta _{\text {SB}}(\overline{M}^{2})\mathcal {D}_h^{0}\mathcal {D}_H^{0*}. \end{aligned}$$

On the one hand, this approach requires the separate calculation of purely photonic and non-photonic contributions. On the other hand, it enables the on-shell evaluation of IR-finite integrals and is thus closer to the full result. However, in case of a virtual photino contribution one needs to be careful not to break supersymmetry by treating the photon differently than its superpartner. Thus, the possibility of such a separate treatment of the photon diagrams, whose numerical impact is small in the studied example process, should be considered in view of the investigated model and its particle content.

### Interference weight factors at 1-loop order

In the previous section, we derived how to include virtual and real contributions in the product of factorised matrix elements in a UV- and IR-finite way. However, special attention is needed to ensure the correct treatment of the on-shell matrix elements of the interference contribution.

We now discuss additional approximations with which the *R*-factor method introduced in Sect. 3.3 can be extended beyond the tree level. We develop a method that facilitates an approximation of the interference term based on higher-order cross sections and decay widths, but only tree level couplings. This technically simpler treatment comes at the price of the further assumption, as in the tree level version of the interference weight factor, that both Higgs masses be equal. Thus, the method presented in this section is an optional, additional approximation with respect to Eq. (105).

Under the assumption of equal masses, the product of unsquared matrix elements for the production and decay of *h* and *H* can be re-expressed at the tree level in terms of either *h* or *H* with the help of Eq. (37). Hence, one can choose to keep the 1-loop matrix elements and to replace only the tree level ones so that only lowest-order couplings will be present in the *x*-factor. We will now apply this prescription to the third term in Eq. (105) containing the 1-loop virtual corrections to the interference term $\text {Int}^{v}$:

121 $$\begin{aligned} \text {Int}^{v}&=2\text {Re}[\lbrace (\mathcal {P}_h^{1}\mathcal {D}_h^{0}+ \mathcal {P}_h^{0}\mathcal {D}_h^{1}) \mathcal {P}_H^{0*} \mathcal {D}_H^{0*} \nonumber \\&\quad + \mathcal {P}_h^{0}\mathcal {D}_h^{0} (\mathcal {P}_H^{1*} \mathcal {D}_H^{0*}+\mathcal {P}_H^{0*} \mathcal {D}_H^{1*}) \rbrace \Delta _h^{\text {BW}}\Delta _H^{\text {BW}*}]\nonumber \\&\simeq 2\text {Re}[(\mathcal {P}_h^{1}\mathcal {D}_h^{0}+ \mathcal {P}_h^{0}\mathcal {D}_h^{1}) \mathcal {P}_h^{0*} \mathcal {D}_h^{0*}\cdot \frac{C_{P_H}^{0*}}{C_{P_h}^{0*}} \frac{C_{D_H}^{0*}}{C_{D_h}^{0*}}\nonumber \\&\quad \cdot \Delta _h^{\text {BW}}\Delta _H^{\text {BW}*}]+2\text {Re}[\lbrace \mathcal {P}_H^{0}\mathcal {D}_H^{0}\cdot \frac{C_{P_h}^{0}}{C_{P_H}^{0}} \frac{C_{D_h}^{0}}{Cc_{D_H}^{0}} (\mathcal {P}_H^{1*} \mathcal {D}_H^{0*}\nonumber \\&\quad +\mathcal {P}_H^{0*} \mathcal {D}_H^{1*}) \Delta _h^{\text {BW}}\Delta _H^{\text {BW}*}\rbrace ^{*}]\nonumber \\&=2\text {Re}[(\mathcal {P}_h^{1}\mathcal {P}_h^{0*}|\mathcal {D}_h^{0}|^{2} + |\mathcal {P}_h^{0}|^{2}\mathcal {D}_h^{1}\mathcal {D}_h^{0*}) x_h^{0}\cdot \Delta _h^{\text {BW}}\Delta _H^{\text {BW}*}] \nonumber \\&\quad +2\text {Re}[(\mathcal {P}_H^{1}\mathcal {P}_H^{0*}|\mathcal {D}_H^{0}|^{2} + |\mathcal {P}_H^{0}|^{2}\mathcal {D}_H^{1}\mathcal {D}_H^{0*}) x_H^{0}\nonumber \\&\quad \,\,\cdot \Delta _H^{\text {BW}}\Delta _h^{\text {BW}*}]. \end{aligned}$$

Hence we exploited the choice of expressing the product of *h*- and *H*-matrix elements either in a weighted sum of both or in terms of one of them. The latter choice, as selected in Eq. (121), has the advantage that the matrix elements containing loop contributions of *h* and only tree level contributions of *H* are transformed in terms of *h* and vice versa. Including the flux factor and the phase space integrals as in Eq. (35), adding soft bremsstrahlung according to the last line of Eq. (105) and keeping in mind that

122 $$\begin{aligned}&\frac{1}{F}\int \mathrm{d}\Phi _{P} 2\text {Re}[\mathcal {P}_i^{1}\mathcal {P}_i^{0*}] = \sigma _{P_i}^{1}, \nonumber \\&\frac{1}{2M_i}\int \mathrm{d}\Phi _{D} (2\text {Re}[\mathcal {D}_i^{1}\mathcal {D}_i^{0*}]+\delta _{{\text {SB}}}|\mathcal {D}_i^{0}|^{2}) = \Gamma _{D_i}^{1}, \end{aligned}$$

the expressions from Eq. (121) lead to

123 $$\begin{aligned} \sigma _\mathrm{{int}}^{1,R} = \frac{\sigma _{P_h}^{1}\Gamma _{D_h}^{0}+\sigma _{P_h}^{0}\Gamma _{D_h}^{1}}{\Gamma _h^\mathrm{{tot}}} \tilde{R}_h +\frac{\sigma _{P_H}^{1}\Gamma _{D_H}^{0}+\sigma _{P_H}^{0}\Gamma _{D_H}^{1}}{\Gamma _H^\mathrm{{tot}}} \tilde{R}_H , \end{aligned}$$

where $\tilde{R}_i$ has been defined in Eq. (41). Equation (123) is meant for the consistent comparison with the full result in the strict one-loop expansion. Using the most precise predictions of all components and the unfactorised tree level result leads to the final prediction:

124 $$\begin{aligned} \sigma _{R}^\mathrm{{best}}= & {} \sigma _\mathrm{{full}}^{0}\!+\!\sum _{i=h,H}(\sigma _{P_i}^{\text {best}}\text {BR}_i^{\text {best}}\!-\!\sigma _{P_i}^{0}\text {BR}_i^{0})\!+\! \sigma _{R}^\mathrm{{int}1}+\sigma _{R}^\mathrm{{int}+},\nonumber \\ \end{aligned}$$

125 $$\begin{aligned} \sigma _{R}^\mathrm{{int}1}= & {} (\sigma _{P_h}^{1}\text {BR}_h^{0}+\sigma _{P_h}^{0}\text {BR}_h^{1})\tilde{R}_h +(\sigma _{P_H}^{1}\text {BR}_H^{0}+\sigma _{P_H}^{0}\text {BR}_H^{1})\tilde{R}_H,\nonumber \\ \end{aligned}$$

126 $$\begin{aligned} \sigma _{R}^\mathrm{{int}+}= & {} \frac{1}{2}\sigma _{P_h}^{1}(\text {BR}_h^{1}\tilde{R}_h+\text {BR}_H^{1}\tilde{R}_{hH}) \nonumber \\&+\frac{1}{2}\sigma _{P_H}^{1}(\text {BR}_H^{1}\tilde{R}_H+\text {BR}_h^{1}\tilde{R}_{Hh}), \end{aligned}$$

where $\sigma _{R}^\mathrm{{int}1}$ denotes the contribution to the interference term for which the product of production cross sections and partial decay widths is restricted to the 1-loop level, but the branching ratios are at all levels normalised to the 2-loop total width from FeynHiggs [48–51]. In addition, $\sigma _{R}^\mathrm{{int}+}$ contains terms beyond the 1-loop level. In Eq. (126), we introduced the generalised interference weight factors $\tilde{R}_{ij}$,

127 $$\begin{aligned} \tilde{R}_{ij} =2M_j\Gamma _j\text {Re}\lbrace x_{ij} I\rbrace , \end{aligned}$$

involving the scaling factors $x_{ij}$,

128 $$\begin{aligned} x_{ij} =\frac{C_{P_h}C_{P_H}^{*}C_{D_h}C_{D_H}^{*}}{|C_{P_i}|^{2}|C_{D_j}|^{2}}, \end{aligned}$$

to account for the product of 1-loop production and decay matrix elements in Eq. (108). For the most precise prediction, the 1-loop branching ratios in Eqs. (125, 126) can additionally be replaced by $\text {BR}_i^\mathrm{{best}}-\text {BR}_i^{0}$ which is beyond the $\mathcal {M}$-method in Eq. (108). As in Eq. (109) for the $\mathcal {M}$-method, the products of tree level production cross section and branching ratios have to be subtracted because their contribution is already accounted for by $\sigma _{\text {full}}^{0}$. The most precise branching ratios can be obtained from FeynHiggs [48–51] including full 1-loop and leading 2-loop corrections.

## Full 3-body decay at the one-loop level

The numerical validation of the gNWA at the next-to-leading order requires the calculation of the process $\tilde{\chi }_4^0\rightarrow \tilde{\chi }_{1}^{0}\,\tau ^{+}\tau ^{-}$ with intermediate *h* and *H* as the full 3-body decay including virtual and real corrections.

Reference [63] provides a 1-loop calculation of the decay of the next-to-lightest neutralino $\tilde{\chi }_2^{0}$ into $\tilde{\chi }_{1}^{0}$ and a pair of leptons, thus a similar process, but with a dominant contribution from an on-shell slepton, while the Higgs propagators are treated as non-resonant. In the following, we focus on the diagrams contributing to resonant intermediate Higgs bosons, as well as box-diagrams with and without Higgs bosons. The 1-loop integrals are computed with LoopTools [43, 80].

### Contributing diagrams

#### Virtual corrections at the neutralino-Higgs vertex

**Fig. 6:**
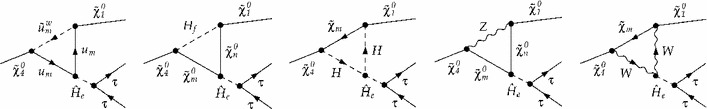
Example triangle diagrams of the 3-body decay $\tilde{\chi }_4^0\rightarrow \tilde{\chi }_{1}^{0}\tau ^{+}\tau ^{-}$ with 1-loop corrections at the $\tilde{\chi }_4^0\tilde{\chi }_{1}^{0}\hat{H}_e$-vertex, where $\hat{H}_e$ denotes a Higgs boson mixed by $\hat{\mathbf{Z }}$-factors, $H_f$ an internal Higgs boson (see text) and $H\equiv H^{\pm }$. *u* and $\tilde{u}$ represent the up-type (s)quarks, $\tilde{\chi }^{0}$ are the neutralinos and $\tilde{\chi }$ the charginos

Virtual SM and MSSM particles contribute to the correction of the $\tilde{\chi }_i^{0}\tilde{\chi }_j^{0}h_k$-vertex. A selection of diagrams is displayed in Fig. 6. We treat here the intermediate Higgs bosons $\hat{H}_e$ appearing outside of the vertex loop contribution as “external”, while $H_f$ denotes an internal Higgs boson within the loop ($e,f=1,2,3$). Furthermore, $H\equiv H^{\pm }$ denotes the charged Higgs bosons. The neutralinos are labelled by $\tilde{\chi }^{0}_n,~n=1,2,3,4$ and the charginos by $\tilde{\chi }_m,~m=1,2$. The first example diagram contains up-type quarks and a squark of generation $m=1,2,3$.

For $\hat{H}_e$ the mixing with $\hat{\mathbf{Z }}$-factors is taken into account, i.e., Eq. (45) is applied for both vertices of $\hat{H}_e$. This treatment has been applied in order to enable a comparison with the factorised production and decay contributions in the gNWA. The appearance of $\hat{\mathbf{Z }}$-factors in external Higgs boson lines is related to the fact that we use a renormalisation scheme without on-shell conditions for the Higgs-boson fields. In such a case, like the $\overline{\text {DR}}$ renormalisation of the Higgs fields employed here, the $\hat{\mathbf{Z }}$-factors are introduced to ensure correct on-shell properties of *external* Higgs bosons [81, 82]. In the NWA, the Higgs bosons appear as external particles in the on-shell production and decay, and we therefore treat the intermediate Higgs bosons of resonant propagators in the full 3-body decay in the same way for comparison purposes.

The triangle corrections appearing at the $\tilde{\chi }_i^{0}\tilde{\chi }_j^{0}h_k$-vertex are renormalised by the counterterm

129 $$\begin{aligned} \delta C_{ijk}^{R/L}&=\frac{e}{2c_{W}s_{W}}\delta c^{(*)}_{ijk} + \left( \delta Z_e - \frac{\delta s_{W}}{s_{W}}- \frac{\delta c_{W}}{c_{W}}\right) C_{ijk}^{R/L}\nonumber \\&\quad +\, \frac{1}{2}\sum _{l=1}^{4}(\delta Z_{li}^{R/L}\,C_{ljk}^{R/L}+\delta \bar{Z}_{jl}^{L/R}\,C_{ilk}^{R/L} +\delta Z_{h_kh_l}C_{ijk}^{R/L}) \end{aligned}$$

in the on-shell scheme, see Ref. [64] and references therein. In Eq. (129), $h_l=\left\{ h,H,A,G\right\}$ for $l=1,2,3,4,$ denote the neutral Higgs and Goldstone bosons. The parameters $M_1,~M_2,~\mu$ are related to the choice of the three electroweakinos which are renormalised on-shell and thus define the choice for the on-shell renormalisation scheme for the neutralino-chargino sector, as mentioned in Sect. 4.2. In our scenario, we identify $\tilde{\chi }_{1}^{0}$ as the most bino-like, $\tilde{\chi }_3^0$ as the most higgsino-like and $\tilde{\chi }_4^0$ as the most wino-like state and hence renormalise these three neutralinos on-shell. By this choice of an NNN scheme, we avoid large mass corrections to the remaining neutralino and the charginos. Alternatively, $\tilde{\chi }_2^0$ instead of $\tilde{\chi }_4^0$ could be identified as the most wino-like state because the two corresponding elements in the matrix *N*, which diagonalises the neutralino mass matrix (see Sect. 4.2), have nearly the same magnitude. Thus, this alternative choice would lead to a comparable sensitivity to the three parameters of this sector and thereby also to a stable renormalisation scheme. But since $\tilde{\chi }_4^0$ is involved in our process as an external particle, we prefer to set it on-shell. The 1-loop effect on the 2-body decay widths $\Gamma (\tilde{\chi }_4^0\rightarrow \tilde{\chi }_{1}^{0}h/H)$ is shown in Fig. 12.

#### Virtual corrections at the Higgs- $\tau ^{+}\tau ^{-}$ vertex and real photon emission

**Fig. 7:**
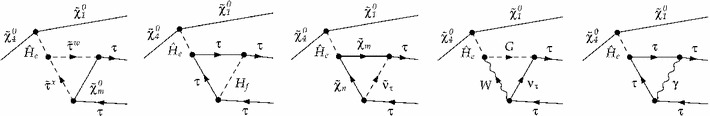
Example triangle diagrams of the 3-body decay $\tilde{\chi }_4^0\rightarrow \tilde{\chi }_{1}^{0}\tau ^{+}\tau ^{-}$ with 1-loop corrections at the $\hat{H}_e\tau ^{+}\tau ^{-}$-vertex, where the particles are labelled as is Fig. 6

Furthermore, the $h_k\tau ^{+}\tau ^{-}$-vertex diagrams shown in Fig. 7 are UV-divergent, and the last diagram is also IR-divergent due to the virtual photon. The UV-divergences are cancelled by the counterterm, analogous to the SM, $\delta C_{h_k\tau ^{+}\tau ^{-}} = \delta C_{h_k\tau \tau }^L\omega _L+\delta C_{h_k\tau \tau }^R\omega _R$, with [75, 83]

130 $$\begin{aligned} \delta C_{h_k\tau ^{+}\tau ^{-}}^{L/R}&= C_{h_k\tau ^{+}\tau ^{-}}^\mathrm{{tree}}\cdot \left( \delta Z_e+\frac{1}{2}\delta Z_{h_k h_k}+\frac{1}{2}\delta Z_{hH}\frac{C_{h_l \tau \tau }^\mathrm{{tree}}}{C_{h_k \tau \tau }^\mathrm{{tree}}}\right. \nonumber \\&\quad \left. -\,\frac{\delta M_W^{2}}{2M_W^{2}} -\frac{\delta s_{W}}{s_{W}} + {{s}_{\beta }}^{2}\delta t_{\beta }\right. \nonumber \\&\quad \left. +\,\frac{\delta m_{\tau }}{m_{\tau }}+\frac{1}{2}\lbrace \delta Z_{\tau }^{L/R}+\delta Z_{\tau }^{R/L\dagger }\rbrace \right) , \end{aligned}$$

where $k,l=h,H$ and $\delta Z_{\tau }^{L/R}$ are the left-/right-handed field renormalisation constants of the $\tau$-lepton. The tree-level couplings $C_{h_k\tau ^{+}\tau ^{-}}^\mathrm{{tree}}$ are given in Eq. (57). The IR-divergent terms vanish for squared matrix elements in the combination of virtual corrections containing a photon in the loop with real photons emitted as soft bremsstrahlung off one of the $\tau$-leptons. Soft photons are defined by the energy cut-off $E_\mathrm{{soft}}^\mathrm{{max}}$. As a prescription for the energy cut-off we use here a fraction of the mass of the decaying particle, namely $E_{\gamma }\le E_\mathrm{{soft}}^\mathrm{{max}}=0.1m_{\tilde{\chi }_4^0}$. All photons below this energy are considered as soft so that they are described by the soft photon factor $\delta _{\text {SB}}$ multiplying the tree level result,

131 $$\begin{aligned} \Gamma _{{\text {SB}}} = \delta _{\text {SB}}\,\Gamma ^{\text {tree}}. \end{aligned}$$

We use the result for $\delta _{\text {SB}}$ of Ref. [75] implemented in FormCalc [43–47]. More details on the separation of soft and hard, collinear and non-collinear QED corrections for this process can be found in Ref. [63].

#### Self-energies involving mixing of neutral bosons

**Fig. 8:**
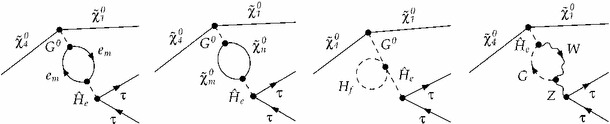
Example self-energy diagrams contributing to the 3-body decay $\tilde{\chi }_4^0\rightarrow \tilde{\chi }_{1}^{0}\tau ^{+}\tau ^{-}$ with 1-loop corrections to the Higgs propagator which mixes with the neutral Goldstone boson *G* and the *Z*-boson. As in Fig. 6, $\hat{H}_e$ denotes a $\hat{\mathbf{Z }}$-mixed neutral Higgs boson and $H_f$ an internal Higgs boson

The diagrams with self-energy corrections of the intermediate (“external”) Higgs boson $\hat{H}_e$ are classified in two categories. On the one hand, there are the mixing contributions between the three neutral Higgs bosons (reduced to $2\times 2$ mixing in case of real MSSM parameters). They are approximated by the $\hat{\mathbf {Z}}$-factors, which were checked to accurately reproduce the full Higgs propagator mixing close to the complex pole (see Sect. 4.1; Refs. [10, 54]). Consequently, no explicit propagator corrections with Higgs self-energies are included. With the $\hat{\mathbf {Z}}$-factors, the strict one-loop order is extended to take more precise mixing effects in the Higgs sector into account. On the other hand, the $\hat{\mathbf{Z }}$-factors do not contain mixing with other neutral particles. Hence, the propagator corrections of a Higgs with the neutral Goldstone boson *G* and the *Z*-boson are calculated explicitly. Some example diagrams are shown in Fig. 8. However, in case of $\mathcal {CP}$-conservation, the mixing between *h* / *H* and *G* / *Z* vanishes.

#### Box diagrams

**Fig. 9:**
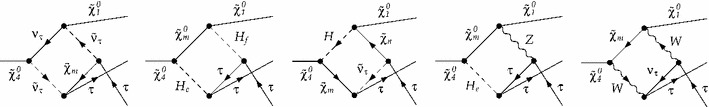
Example box diagrams of the 3-body decay $\tilde{\chi }_4^0\rightarrow \tilde{\chi }_{1}^{0}\tau ^{+}\tau ^{-}$ (with and without Higgs bosons), where the particles are labelled as is Fig. 6. Only internal Higgs bosons $H_f$ appear in the loop

Finally, the $\tilde{\chi }_4^0$ cannot only decay into $\tilde{\chi }_{1}^{0}\tau ^{+}\tau ^{-}$ via a resonant Higgs boson, but also through box diagrams. Figure 9 depicts some example diagrams with and without Higgs bosons. No counterterms are necessary because the boxes are UV-finite by themselves. The box diagrams are explicitly calculated including the full MSSM spectrum in the loops, but, as expected, those non-resonant contributions are found to be numerically suppressed. This is important for the comparison with the gNWA at the 1-loop level in Sect. 9.2 since the boxes cannot be factorised.

### Comparison of the tree level and 1-loop result

**Fig. 10:**
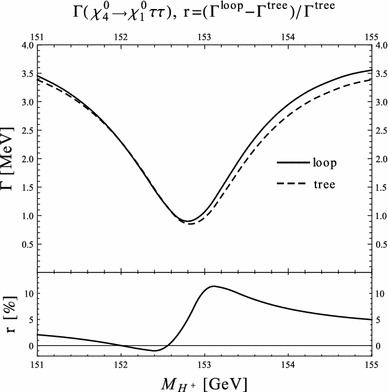
The 1$\rightarrow$3 decay width $\Gamma (\tilde{\chi }_4^0\rightarrow \tilde{\chi }_{1}^{0}\tau ^{+}\tau ^{-})$. *Upper panel* Tree-level mediated by resonant *h*, *H* including their interference (*dashed*) and full 1-loop result with vertex, soft photon and propagator corrections to the resonant *h*, *H*-exchange and, in addition, non-resonant box contributions (*solid*), both supplemented by higher-order Higgs masses, total widths and $\hat{\mathbf{Z }}$-factors. *Lower panel* Relative loop contribution $r=(\Gamma ^{\text {loop}}-\Gamma ^{\text {tree}})/\Gamma ^{\text {tree}}$ in percent

Figure 10 shows the resulting decay width of $\tilde{\chi }_4^0$ into $\tilde{\chi }_{1}^{0}$ and a $\tau ^{+}\tau ^{-}$-pair as the full 3-body decay. As mentioned in Sect. 6.2, the *Z*-, *A*-, *G*- and slepton-exchange is not included in this section, but the interference between all other contributions to the 3-body decay is taken into account. The tree-level and 1-loop results are based on the product of $\hat{\mathbf{Z }}$-factors and Breit–Wigner propagators with higher-order Higgs masses, total widths and $\hat{\mathbf{Z }}$-factors. Despite being an approximation of the complete Higgs propagator mixing, see Eq. (49), it is here referred to as the full result that will consistently serve as a reference for the validation of the gNWA at the 1-loop level.

The full 1-loop decay width includes the vertex corrections at the production and the decay vertex and box contributions as well as self-energy corrections to the propagator and bremsstrahlung off the $\tau$-leptons in the final state.

The NLO decay width (solid) is enhanced relative to the LO result (dashed) in most of the analysed parameter interval, up to $11\,\%$, as the plot of the ratio $r=(\Gamma ^{\text {loop}}-\Gamma ^{\text {tree}})/\Gamma ^{\text {tree}}$ shows. However, around $M_{H^{\pm }}\simeq 152$ GeV, the 1-loop corrections vanish.

## Numerical validation of the gNWA at the loop level

In this example, the calculation of the full process at the 1-loop level is still manageable, where *full* here means the 3-body decays with Breit–Wigner propagators and $\hat{\mathbf{Z }}$-factors, though without the *Z*-, *A*- and *G*-boson exchange. But we aim at validating the generalised narrow-width approximation at the 1-loop level so that it can be applied on kinematically more complicated processes for which the factorisation into production and decay is essential to enable the computation of higher order corrections.

Our strategy is to combine the NLO corrections for the production and decay subprocesses in such a way that the gNWA prediction can be consistently compared to the full 1-loop calculation. Only the box diagrams are left out in the gNWA compared to the 3-body decays.

### 2-body decays

**Fig. 11:**
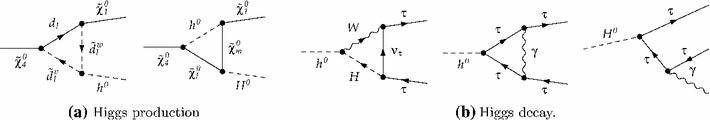
Example diagrams of the 2-body decays for **a** Higgs production in $\tilde{\chi }_4^0\rightarrow \tilde{\chi }_{1}^{0}h/H$ at NLO and **b** Higgs decay in $h/H\rightarrow \tau ^{+}\tau ^{-}$ at NLO with virtual and real corrections

**Fig. 12:**
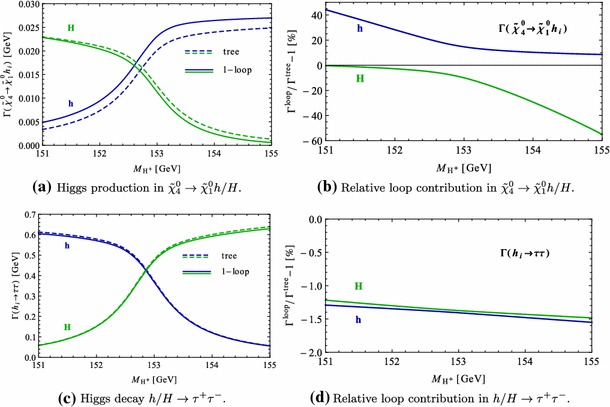
2-body decay widths of **a** $\tilde{\chi }_4^0\rightarrow \tilde{\chi }_{1}^{0}h_i$ and **c** $h_i\rightarrow \tau ^{+}\tau ^{-}$ with $h_i=h$ (*blue*) and *H* (*green*) at the tree level (*dashed*) or at the 1-loop level (*solid*), and the relative effect of the loop contributions (**b**), (**d**)

The gNWA at NLO requires the 1-loop contributions to the 2-body decays as subprocesses. For the production, we calculate the full 1-loop corrections to $\Gamma (\tilde{\chi }_4^0\rightarrow \tilde{\chi }_{1}^{0}h/H)$ in the NNN on-shell renormalisation scheme, see Refs. [56, 64, 65], with the same choice of on-shell states as in the 3-body-decay described in Sect. 8.1.1. Higgs mixing is taken into account by $\hat{\mathbf{Z }}$-factors, but mixing with *G*-/*Z*-bosons is generated explicitly, which, however, vanishes in this $\mathcal {CP}$-conserving scenario. Some example diagrams for vertex corrections are shown in Fig. 11a. Figure 12a presents the resulting 2-body decay widths for the production of *h* (blue) and *H* (green) at the tree level (dashed) and the 1-loop level (solid). While the 1-loop corrections increase $\Gamma (\tilde{\chi }_4^0\rightarrow \tilde{\chi }_{1}^{0}h)$, they decrease the production of *H* from the decay of $\tilde{\chi }_4^0$. The substantial relative effect can be seen in Fig. 12c.

For the decay, the full vertex corrections to $h_i\rightarrow \tau ^{+}\tau ^{-}$ are included. Furthermore, real soft photon emission off the $\tau$-leptons in the final state is included. In order to allow for a meaningful comparison between the gNWA and the full calculation, the energy cut-off is defined by the same value $E_\mathrm{{soft}}^\mathrm{{max}}=0.1m_{\tilde{\chi }_4^0}$ as in the 3-body decay. Example diagrams are displayed in Fig. 11b, where the first diagram belongs to the IR-finite ones, but the second and third diagrams are IR-divergent. The emission of a real photon is not directly calculated as a 3-body decay, but still with the 2-body phase space in the soft-photon approximation. The numerical influence of the corrections of $\mathcal {O}(\alpha )$ on $\Gamma (h_i\rightarrow \tau ^{+}\tau ^{-})$ is shown in Fig. 12c. The 1-loop and real corrections slightly decrease both decay rates (for $h_i=h,H$) by 1.2–$1.5\,\%$ as displayed in Fig. 12d.

### On-shell matrix elements and *R*-factor approximation

The on-shell factorisation of the interference term has already been applied at the leading order in Sect. 6.2. In this section, we will investigate its accuracy at the next-to-leading order. Since a wide range of processes even with many external particles can be computed at lowest order without applying the NWA, we use the full leading order result of the three-body decay (i.e., without NWA) and add the 1-loop contribution for which we use the gNWA. With this procedure, we apply the on-shell approximation only when necessary without introducing an avoidable uncertainty at the tree level.^7^

**Fig. 13:**
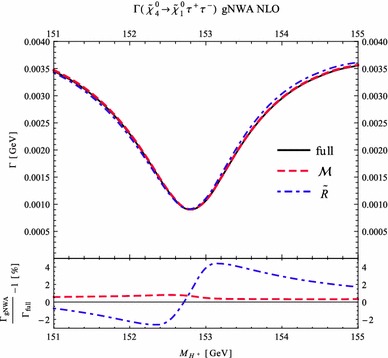
*Upper panel* The decay width $\tilde{\chi }_4^0\rightarrow \tilde{\chi }_{1}^{0}\tau ^{+}\tau ^{-}$ at the 1-loop level with resonant *h*, *H*-exchange and, for the full 3-body decay (*black*, *solid*), with box contributions. The gNWA with on-shell matrix elements is denoted by $\mathcal {M}$ (*red*, *dashed*), and the gNWA with interference weight factors is denoted by $\tilde{R}$ (*blue*, *dash-dotted*). *Lower panel* The relative deviation of the gNWA (matrix element and *R*-factor approximation) from the full 1-loop result in percent

In Fig. 13, we compare the numerical results of the method of on-shell matrix elements using Eqs. (106) and (107), denoted by $\mathcal {M}$, and of the interference weight factor approximation from Eq. (123), denoted by $\tilde{R}$, with the full 1-loop result as calculated in Sect. 9. The upper panel shows the prediction of the partial width $\Gamma (\tilde{\chi }_4^0\rightarrow \tilde{\chi }_{1}^{0}\tau ^{+}\tau ^{-})$. The lines of the gNWA based on matrix elements (red, dashed) and the full 1-loop calculation (black, solid) lie nearly on top of one another. Also the additional $\tilde{R}$-factor approximation (blue, dash-dotted) yields a good qualitative agreement with the full result, but less accurate than achieved by the on-shell matrix elements. The lower panel visualises the relative deviation of the decay width predicted by the two versions of the gNWA from the full result. As expected, the *R*-factor method reproduces the full result best where the difference between $M_h$ and $M_H$ is smallest, i.e., in the centre of the analysed parameter interval. But the assumption of equal masses becomes worse away from the centre of the analysed interval, leading to a deviation from the full 1-loop result of up to $4.5\,\%$. Thus, for those parameters the matrix element method performs clearly better within an accuracy of better than $1\,\%$.

**Fig. 14:**
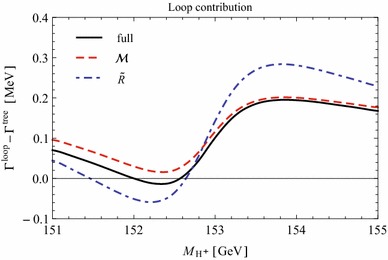
Pure loop contributions in the full calculation (*black*, *solid*) and approximated by the gNWA using the matrix element method denoted by $\mathcal {M}$ (*red*, *dashed*) and using the *R*-factor approximation denoted by $\tilde{R}$ (*blue*, *dash-dotted*)

In order to further investigate how well the gNWA predicts the interference term at the 1-loop level, we take a closer look in Fig. 14 at the pure loop contribution $\Gamma ^{\text {loop,pure}}=\Gamma ^{\text {loop}}-\Gamma ^{\text {tree}}$ of the full three-body decay (black, solid), the gNWA using on-shell matrix elements (red, dashed, denoted by $\mathcal {M}$) and the $\tilde{R}$-factor approximation (blue, dash-dotted, denoted by $\tilde{R}$). While at the tree level we found that both versions of the gNWA work comparably well (see Fig. 5), the $\mathcal {M}$-method provides a significantly better prediction of the interference term at the 1-loop level.

**Fig. 15:**
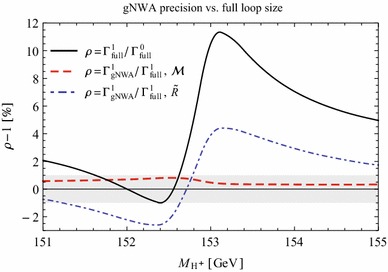
Precision of the gNWA at the 1-loop level using the matrix element method denoted by $\mathcal {M}$ (*red*, *dashed*) and using the *R*-factor approximation denoted by $\tilde{R}$ (*blue*, *dash-dotted*) compared to the relative size of the loop contribution in the full calculation (*black*). The $\pm 1\,\%$ region is indicated in *grey*

When the gNWA is used to approximate one-loop effects, we need to compare the accuracy of the approximation with the overall size of the loop correction. Figure 15 provides a comparison between the precision of the gNWA with respect to the full calculation (for on-shell matrix elements denoted by $\mathcal {M}$ in red and the *R*-factor approximation denoted by $\tilde{R}$ in blue) and the relative size of the 1-loop correction to the 3-body decay width in black. While the loop correction ranges from $-1$ to $11\,\%$ in this example case, the deviation of the matrix element method from the full result remains below $1\,\%$. The uncertainty of this approximation is therefore significantly smaller than the typical size of the loop correction in this case. The deviation of the *R*-factor approximation from the full result is found to be larger, within $-3$–$4.5\,\%$ in this case, but it is still about a factor of two smaller than the size of the loop correction in the region where the latter is sizable.

The plot shows that the overall performance of the gNWA with the $\mathcal {M}$-method is good except for the region around $M_{H^{\pm }}\simeq 152$– 152.5 GeV where the $\mathcal {M}$-method uncertainty exceeds the relative size of the full loop correction slightly. But here the full loop correction is in fact very small. Keeping in mind that the full calculation is subject to uncertainties itself (e.g. from missing higher-order corrections) which might reach the level of $1\,\%$ (for illustration, the $\pm 1\,\%$ range is indicated in the plot), the $\mathcal {M}$-method can be regarded as adequate to approximate loop corrections to the interference term within the expected uncertainty of the full result (as long as non-factorisable corrections remain numerically suppressed). On the other hand, the *R*-factor method gives rise to larger deviations and should therefore be regarded as a simple estimate of the higher-order result including interference effects.

### Separate treatment of photon contributions

**Fig. 16:**
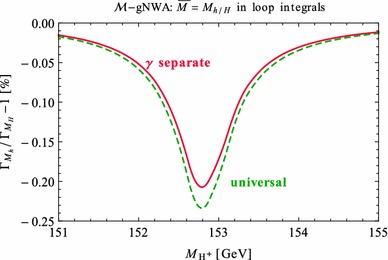
Impact of the dependence of the gNWA on the choice of the mass $\overline{M}$ (see text). The relative deviation between $\Gamma _{M_h}$ and $\Gamma _{M_H}$, where $\Gamma _{M_i}\equiv \Gamma _\mathrm{{gNWA}}^{\mathcal {M}}(\overline{M}^{2}=M_i^{2})$, is shown for the universal treatment of all one-loop matrix elements for the decay and for the case where the photonic contribution is treated separately

As discussed in Sect. 7.1.2, the factor $\delta _{\text {SB}}$, which multiplies the squared tree level matrix element to account for the contribution of soft bremsstrahlung, and the IR-divergent loop integrals must be evaluated at the same mass to enable the cancellation of IR-singularities between real and virtual photon contributions. In order to reduce the ambiguity whether to choose the common mass $\overline{M}=M_h$ or $M_H$, the IR-finite diagrams can be evaluated at their correct mass shell. Figure 16 compares the dependence of the gNWA result on the ambiguous mass choice, i.e., the relative deviation between $\Gamma _\mathrm{{gNWA}}(\overline{M}=M_h)$ and $\Gamma _\mathrm{{gNWA}}(\overline{M}=M_H)$, for the matrix element method. The dashed green line represents the universal treatment where the loop integrals in all decay one-loop matrix elements are evaluated at $\overline{M}^{2}$ whereas the solid red line shows the separate calculation of the photonic contribution as described in Sect. 7.1.2. The impact of the dependence of the gNWA on the choice of the mass $\overline{M}$ is found to be rather small, giving rise to a maximum deviation of $0.23\,\%$ for the universal treatment of all one-loop matrix elements for the decay. Restricting this approximation just to the photonic contribution is seen to have an insignificant effect in this example, reducing the deviation to $0.2\,\%$.

### gNWA prediction with most precise input values

As a first step, we defined the gNWA at the 1-loop order for a consistent comparison between the gNWA and the full 1-loop calculation. As an exception, the Higgs masses, total widths and wave function normalisation factors $\hat{\mathbf{Z }}$ have been obtained from FeynHiggs [48–51] at the 2-loop order and used both in the gNWA and the full calculation. In this section we want to exploit the factorisation and include all components at the highest available precision. This means for the gNWA with the on-shell matrix element method and the *R*-factor approximation that we use the calculated 1-loop production part and the FeynHiggs branching ratios in $\Gamma _{P}(\tilde{\chi }_4^0\rightarrow \tilde{\chi }_{1}^{0}h_i)\cdot \text {BR}_{D}(h_i\rightarrow \tau ^{+}\tau ^{-})$. Furthermore, the product of on-shell matrix elements from Eq. (105) is expanded up to the product of 1-loop matrix elements in Eq. (109). The higher-order extension of the *R*-factor approximation is defined in Eq. (124).

**Fig. 17:**
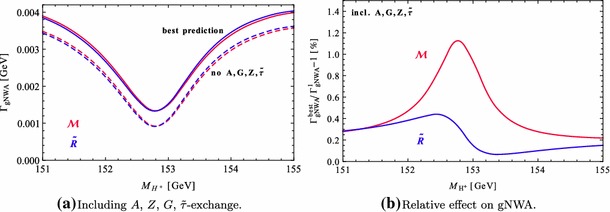
**a** The gNWA using the most accurate predictions for all parts of the process, supplemented with a tree-level result with (*solid*) and without (*dashed*) the additional $A,\,G,\,Z$ and $\tilde{\tau }$-exchange contributions, for the $\mathcal {M}$-method (*red*) and the $\tilde{R}$-approximation (*blue*). **b** The relative effect of the most precise branching ratios and the product of 1-loop terms on the prediction of the gNWA with on-shell matrix elements (*red*, denoted by $\mathcal {M}$) and the *R*-factor approximation (*blue*, denoted by $\tilde{R}$)

So far we have neglected additional contributions that do not play a role in the discussion of the interference effects between contributions with *h* and *H* exchange in the decay of $\tilde{\chi }_4^0\rightarrow \tilde{\chi }_{1}^{0}\tau ^{+}\tau ^{-}$ for the considered $\mathcal {CP}$-conserving scenario. In order to obtain a more phenomenological prediction of $\Gamma (\tilde{\chi }_4^0\rightarrow \tilde{\chi }_{1}^{0}\tau ^{+}\tau ^{-})$ we now take into account also the resonant exchange of the $\mathcal {CP}$-odd Higgs boson *A*, the neutral Goldstone boson *G* and the *Z*-boson, as well as the non-resonant 3-body decay via a $\tilde{\tau }$. We include the contributions from $A,\,G,\,Z$ and $\tilde{\tau }$-exchange at the tree-level, while at the loop level we incorporate the most precise gNWA result (where those additional contributions are neglected). Figure 17a shows the prediction of the higher-order improved gNWA, supplemented by the full tree-level contribution including $A,\,G,\,Z$ and $\tilde{\tau }$-exchange diagrams, as solid lines using on-shell matrix elements (red) and the *R*-factor approximation (blue). The corresponding results where the $A,\,G,\,Z$ and $\tilde{\tau }$-exchange contributions have been neglected are indicated by the dashed lines. The contributions from $A,\,G,\,Z$ and $\tilde{\tau }$ are found to yield a non-negligible upward shift in this example.

Figure 17b shows the impact of including the most precise branching ratios and the product of 1-loop matrix elements in the gNWA, denoted by $\Gamma ^{\text {best}}_{\text {gNWA}}$. For the matrix element method (in red, denoted by $\mathcal {M}$), this amounts to up to $1.2\,\%$ relative to the 1-loop formulation used above for the comparison with the result for the 3-body decay. For the *R*-factor approximation (in blue, denoted by $\tilde{R}$), the effect of up to $0.4\,\%$ is smaller because the effect on the interference term beyond the 1-loop order turns out to be negative. With reference to the gNWA including only *h* and *H*, the relative impact of the higher-order corrections is slightly higher ($1.6\,\%$ for the matrix element method and $0.6\,\%$ for the *R*-factor approximation).

The numerical size of the contributions beyond the 1-loop order depends on the process and scenario, but the gNWA allows for their inclusion also in the interference term.

## Conclusions

In this paper, we have developed a generalisation of the standard narrow-width approximation that extends the applicability of this important tool to scenarios where interference effects between nearly mass-degenerate particles are important. This can be the case in many extensions of the SM where the spectrum of the new particles is such that the mass difference between two or more particles is smaller than one of their total decay widths. In such a case, their resonances overlap so that the interference cannot be neglected if the two states mix. In order to still enable the convenient factorisation of a more complicated process into production and decay of an intermediate particle, we have demonstrated how to factorise also the interference term. This is achieved by evaluating the production and decay matrix elements on the mass-shells of the resonant particles in analogy to the terms present in the standard NWA. If one additionally assumes equal masses of the intermediate particles, it is possible to further approximate the interference contribution by an interference weight factor, *R*, in terms of production cross sections, decay branching fractions, ratios of couplings and a universal, process independent integral over Breit–Wigner propagators.

We have developed this generalised narrow-width approximation both at the tree-level and at one-loop order. Following the analytic derivations, we have discussed the application to a simple example process in the context of the MSSM with real parameters. We have considered the three-body decay of the heaviest neutralino via a resonant neutral, $\mathcal {CP}$-even Higgs boson, *h* or *H*, into the lightest neutralino and a pair of $\tau$-leptons. This process is well-suited for a test of the gNWA since it is sufficiently simple so that the full process can be calculated at the loop level and compared with the predictions of the gNWA. Within the gNWA this process can be decomposed into basic kinematic building blocks, namely two subsequent 2-body decays, and the interference contributions involve only scalar particles. The discussion of interference effects can therefore be disentangled from spin-correlation issues. Furthermore, the process involves charged external particles, so that the issue of the cancellation of IR divergencies between virtual loop corrections and bremsstrahlung contributions is relevant, while the fact that only the final state particles are charged makes the treatment of the IR-divergent contributions very transparent.

We have validated the gNWA at the Born level (supplemented by higher-order Higgs masses, widths and mixing factors) and at the 1-loop level including corrections of $\mathcal {O}(\alpha )$ with respect to the lowest order. Within the considered parameter region, the chosen modified $M_h^\mathrm{{max}}$-scenario leads to a small difference between the loop-corrected masses of $M_h$ and $M_H$ below their total widths. This configuration results in a large negative interference term so that in the standard NWA, where the interference contribution is not taken into account, the 3-body decay width is overestimated by a factor of up to five in this example. Hence, the standard NWA is clearly insufficient in this scenario. The inclusion of the factorised interference term, however, leads to an agreement with the unfactorised decay width within few percent. At the tree level, the method of on-shell matrix elements and the *R*-factor approximation lead to very similar results.

However, at the Born level the methods for calculating multi-leg processes without further approximations are very advanced. Accordingly, a particular interest in the NWA concerns its application to the loop level, where the difficulty in computing processes involving a variety of different mass scales grows very significantly with the number of external legs of the process. In many cases the factorisation into different sub-processes provided by the NWA is essential to enable the computation of higher-order contributions. In cases where a full tree level calculation is feasible, the NWA can therefore be applied just at the loop level in order to facilitate the computation of the higher-order corrections, while the lowest order contributions are evaluated without further approximations in order to avoid an unnecessary theoretical uncertainty.

For a validation of the gNWA beyond the LO we have performed the 1-loop calculation of $\Gamma (\tilde{\chi }_4^0\rightarrow \tilde{\chi }_{1}^{0}\tau ^{+}\tau ^{-})$ including all vertex corrections, self-energies involving Higgs-Goldstone/*Z* mixing, Higgs-Higgs mixing contributions via finite wave function normalisation factors, box diagrams, as well as soft photon radiation. All higher order corrections except for the box diagrams factorise, which makes a separate calculation of the 1-loop production and decay part possible as long as the non-factorisable contributions remain sufficiently small. We have shown that within the gNWA the factorised interference term at the next-to-leading order is both UV- and IR-finite. In order to preserve the cancellations of IR-singularities between virtual and real photon contributions also in the on-shell matrix elements, all IR-divergent integrals in matrix elements and the soft-photon factor were evaluated at the same mass value. This prescription could be further improved by extracting the singular parts from the real photon contribution and applying the NWA only to those terms which match the singularities from the virtual photons. Furthermore, we have extended the interference weight factor to the 1-loop level. In the numerical comparison to the 3-body decay width, the gNWA based on 1-loop on-shell matrix elements agrees with the full 1-loop result within an accuracy of better than $1\,\%$, which is much below the typical size of the loop corrections in this case. The gNWA with interference weight factors, on the other hand, deviates from the full result by up to $4\,\%$, which is still about a factor of two smaller than the size of the loop correction in the region where the latter is sizable. Therefore the method of on-shell matrix elements appears to be a well-suited approach for predicting the interference term at 1-loop order within roughly the remaining theoretical uncertainty of the full result, while the additional *R*-factor approximation may be of interest as a technically simpler rough estimate of the higher-order result including interference effects.

In our discussion we have first focussed on the strict $\mathcal {O}(\alpha )$ contribution relative to the lowest order within the gNWA (except for masses, total widths and wave function normalisation factors, for which we have incorporated dominant 2-loop contributions throughout this work) for the purpose of a consistent comparison with the 3-body decay width. In the most accurate final result the factorisation into subprocesses for production and decay has the virtue that higher-order corrections can naturally be implemented into each of the subprocesses, which formally corresponds to a higher-order effect for the full process. This applies also to the interference term, where we have discussed the incorporation of higher-order contributions for the two considered versions of the gNWA.

While much of our discussion has been directed to the specific example process that we have investigated, we have provided a generic formulation of the gNWA and we have commented on various features that are relevant for more complicated processes. The method presented here should therefore be transferable to processes with more external legs, with a more complicated structure of IR divergencies, and to cases where the interference arises between particles of non-zero spin.

Based on the methodical study presented here, a next step will be a more detailed investigation of phenomenological applications of the gNWA. This will be addressed in a forthcoming publication.

## Footnotes

1 We refer to partonic cross sections, but the overall $q^{2}$-dependence can be affected by folding with pdfs.

2 We used FeynArts-3.7, FormCalc-7.4, LoopTools-2.8 and FeynHiggs-2.9.3.

3 These helicity matrix elements correspond to the FormCalc-HelicityMEs via $A_{ij} = 4\cdot \text {MatF}(i,j)$. The factor of 4 arises because the FormCalc expressions are multiplied later on by 2 for each external fermion.

4 In this section, the *full* tree level refers to the sum of *h*- and *H*-mediated 3-body decays including the interference term (but without *A*- and *Z*-boson exchange or non-resonant propagators) at the improved Born level, i.e. including Higgs masses, total widths and $\hat{\mathbf{Z }}$-factors at the leading 2-loop level from FeynHiggs [48–51].

5 If the full Born cross section cannot be calculated, this term can be replaced by the gNWA at the Born level.

6 With *strict 1-loop* we refer to the expansion of the products of matrix elements whereas 2-loop Higgs masses, total widths and wave function renormalisation factors are employed.

7 As a further step, one could split the real photon contribution into IR-singular and finite terms and apply the NWA only on the singular ones according to Refs. [4, 37].
